# *In vitro* and intracellular activities of novel thiopeptide derivatives against macrolide-susceptible and macrolide-resistant *Mycobacterium avium* complex

**DOI:** 10.1128/spectrum.01825-23

**Published:** 2023-08-18

**Authors:** Jiyun Park, Lee-Han Kim, Ju Mi Lee, Sangwon Choi, Young-Jin Son, Hee-Jong Hwang, Sung Jae Shin

**Affiliations:** 1 Department of Microbiology, Institute for Immunology and Immunological Disease, Brain Korea 21 Project for Graduate School of Medical Science, Yonsei University College of Medicine, Seoul, South Korea; 2 A&J Science Co., Ltd, Daegu, South Korea; University of California, San Diego, La Jolla, California, USA

**Keywords:** *Mycobacterium avium *complex, micrococcin p2, thiopeptide derivatives, minimum inhibitory concentration, intracellular activity, macrolide-resistant MAC

## Abstract

Unsatisfactory outcomes following long-term multidrug treatment in patients with *Mycobacterium avium* complex (MAC) pulmonary disease have urged us to develop novel antibiotics. Thiopeptides, a class of peptide antibiotics derived from natural products, have potential as drug candidates that target bacterial ribosomes, but drug development has been hampered due to their extremely poor solubility. Here, we evaluated three new compounds (AJ-037, AJ-039, and AJ-206) derived from the thiopeptide micrococcin P2 with enhanced aqueous solubility; the derivatives were generated based on structure-activity relationship analysis. We conducted *in vitro* drug susceptibility and intracellular antimycobacterial activity testing of the three thiopeptide derivatives against various MAC strains, including macrolide-resistant MAC clinical isolates. These compounds showed low MICs against MAC, similar to that of clarithromycin (CLR). In particular, two compounds, AJ-037 and AJ-206, had intracellular antimycobacterial activities, along with synergistic effects with CLR, and inhibited the growth of MAC inside macrophages. Moreover, these two compounds showed *in vitro* and intracellular anti-MAC activities against macrolide-resistant MAC strains without showing cross-resistance with CLR. Taken together, the results of the current study suggest that AJ-037 and AJ-206 can be promising anti-MAC agents for the treatment of MAC infection, including for macrolide-resistant MAC strains.

**IMPORTANCE:**

Novel antibiotics for the treatment of MAC infection are urgently needed because the treatment outcomes using the standard regimen for *Mycobacterium avium* complex (MAC) pulmonary disease remain unsatisfactory. Here, we evaluated three novel thiopeptide derivatives (AJ-037, AJ-039, and AJ-206) derived from the thiopeptide micrococcin P2, and they were confirmed to be effective against macrolide-susceptible and macrolide-resistant MAC. Our thiopeptide derivatives have enhanced aqueous solubility through structural modifications of poorly soluble thiopeptides. The thiopeptide derivatives showed minimal inhibitory concentrations against MAC that were comparable to clarithromycin (CLR). Notably, two compounds, AJ-037 and AJ-206, exhibited intracellular antimycobacterial activities and acted synergistically with CLR to hinder the growth of MAC within macrophages. Additionally, these compounds demonstrated *in vitro* and intracellular anti-MAC activities against macrolide-resistant MAC strains without showing any cross-resistance with CLR. We believe that AJ-037 and AJ-206 can be promising anti-MAC agents for the treatment of MAC infections, including macrolide-resistant MAC strains.

## INTRODUCTION

Nontuberculous mycobacteria (NTM) are opportunistic pathogens that include mycobacterial species other than the *Mycobacterium tuberculosis* complex and *Mycobacterium leprae*. NTM are ubiquitous organisms in environments such as soil, water, and dust. Over 190 NTM species and subspecies have been identified to date, and some of these cause NTM infectious diseases in humans (1). The annual prevalence and mortality of NTM infection have gradually increased worldwide, especially in developed countries (2 – 4).

*Mycobacterium avium* complex (MAC), mainly consisting of *Mycobacterium avium* and *Mycobacterium intracellulare*, is the most common cause of chronic pulmonary disease (PD) in humans among NTM species (5 – 8). The recommended standard treatment regimen consists of macrolides (clarithromycin (CLR) and azithromycin) with ethambutol (EMB) and rifampicin (RIF) over 12 months after culture conversion (9 – 11). Although the standard regimen based on macrolides, a core drug, has been established, the culture conversion rates are up to 50–65% and even lower with the development of macrolide resistance (12 – 14). Thus, more effective antimicrobials for the treatment of MAC-PD are urgently needed.

Thiopeptides have been identified as a family of antibacterial agents with a wide range of other interesting biological activities (15 – 17). Micrococcin P (MP) is the first thiopeptide antibiotic derived from natural products that are composed of a mixture of MP1 and MP2 (18 – 20). Their potent antibacterial activity was attributed to tight binding to a specific region of bacterial ribosomes. Specifically, their mode of action differs depending on the size of the macrocyclic cores. For instance, those that have 26-membered rings, such as MP2, bind tightly to a cleft between the 23S ribosomal RNA subunit and the L11 domain, resulting in the blockage of the binding region for elongation factor G (EF-G) (21, 22). Those with 29-membered macrocycles bind strongly to the thermo unstable elongation factor known as the EF-Tu factor (23). In either case, essential bacterial protein synthesis is halted. In some studies, thiopeptides are not only active against gram-positive bacteria, including antibiotic-resistant bacteria, but also have anti-plasmodial, anti-fungal, anti-virus, and anti-cancer activities (16, 24 – 28). In addition, MP1 exhibited bactericidal activity against *M. tuberculosis* without causing any cytotoxicity to human cells or cross-resistance to anti-TB drugs (18, 29). Therefore, thiopeptides have attracted attention as ideal candidates for the development of a new antibiotic class because they have low toxicity and do not cause cross-resistance by interacting with various antibiotics (30).

In contrast to antimicrobial research on MP1, little is known about the antibiotic effect of MP2 against a variety of bacteria. Recently, thiopeptide MP2 has been reported to have an antimicrobial effect against *Clostridioides difficile*, including clinical isolates. It also has pharmacokinetic properties that make it particularly promising and a mode of action that is distinct from other antibiotics (31). Furthermore, AJ-024, a nitroimidazole derivative of a 26-membered thiopeptide based on MP2, was proven to be a promising anti*-C*. *difficile* lead compound (32). Thus, MP2 could be a valuable platform for the development of new antibiotics. However, the notoriously poor solubility of thiopeptides, including MP1 and MP2, is a major drawback in clinical development (33, 34). For this reason, medicinal chemistry campaigns aimed at improving aqueous solubility and pharmacokinetic properties have identified three lead compounds (AJ-037, AJ-039, and AJ-206) based on MP2. Briefly, the biosteric displacement of the ethylidene unit with cyclopropane was attempted. All of these compounds were synthesized by A&J Science Co, Ltd. (Daegu, South Korea) (35, 36).

In this study, we assessed the antimycobacterial potential of three thiopeptide derivatives, AJ-037, AJ-039, and AJ-206, for the first time against MAC clinical isolates, including macrolide-resistant MAC strains.

## RESULTS

### Characteristics of derivatives based on MP2

**Fig 1:**
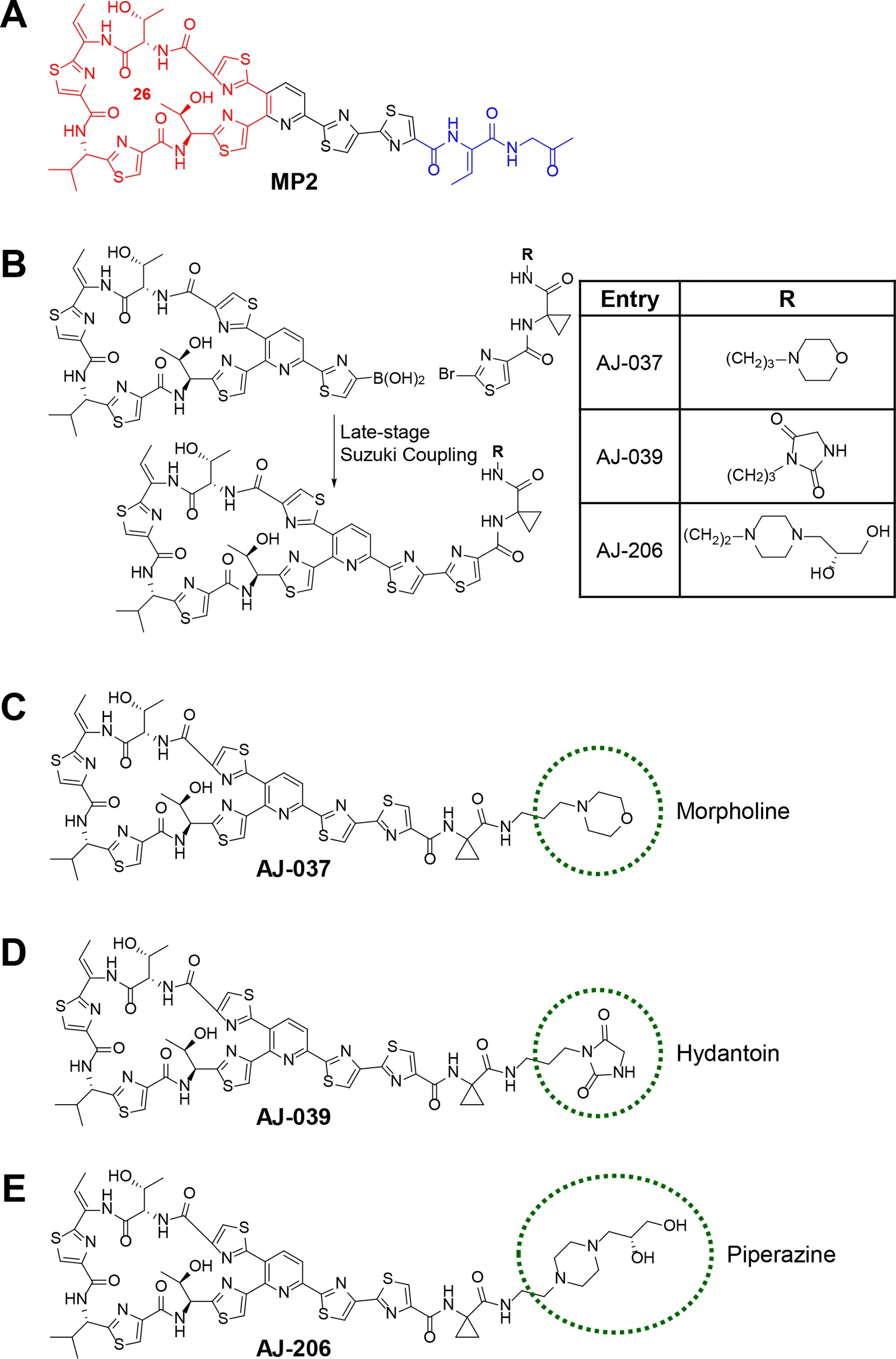
Chemical structure of MP2 and thiopeptide derivatives. Chemical structures of (**A**) MP2, (**C**) AJ-037, (**D**) AJ-039, and (**E**) AJ-206. The red scaffolds are conserved for activity properties. The blue scaffolds were modified to optimize the aqueous solubility of MP2. (**B**) The union of boronic acid with the corresponding 2-bromothiazole was realized by the late Suzuki coupling reaction. (**C**) AJ-037, (**D**) AJ-039, and (**E**) AJ-206 underwent chemical modification through the use of distinct derivatives, specifically morpholine, hydantoin, and piperazine. Changed scaffolds are marked in green.

Using our previous synthetic work on MP2, we revealed that structural modifications of the macrocyclic ring resulted in diminished activity by affecting the mode of action (marked in red) (Fig. 1A). Additionally, the structural modifications were permissible only at the level of the eastern bis-thiazole side chain (marked in blue) (Fig. 1A). Thus, we have directed our attention to optimizing MP2’s aqueous solubility through the incorporation of polar substituents bearing saturated nitrogen heterocycles. All of these compounds were synthesized via a late-stage Suzuki coupling in which boronic acid is combined with the corresponding 2-bromothiazole (Fig. 1B). These nitrogen-containing heterocycles are known to harbor distinctive advantages due to their hydrogen bonding ability. Moreover, their basic functional group could form an appropriate salt that could further enhance aqueous solubility. These efforts have culminated in a few lead compounds, such as AJ-037, AJ-039, and AJ-206 (Fig. 1C through E). AJ-037, AJ-039, and AJ-206 have structural modifications to the morpholine derivative, hydantoin derivative, and piperazine derivative, respectively (marked in green) (Fig. 1C through E). A full account of the structural characterizations (^1^H NMR, ^13^C NMR, HPLC traces and high-resolution mass spectrometry (HRMS)) of AJ-037, AJ-039, and AJ-206 is provided in Fig. S1 to S3 in the supplemental material. Additionally, the solubility profile of these compounds is shown in Table 1. In particular, the preparation of the corresponding HCl salt further enhanced their solubility profile. In the case of AJ-206, the solubility was increased by a factor of over 140,000-fold compared to that of MP2. Thus, the widely considered poor solubility of the thiopeptide has been overcome by the introduction of nitrogen heterocycles and the formation of the corresponding salt.

**TABLE 1:** Aqueous solubility of MP2 and thiopeptide derivatives

Chemical	Solubility pH 7.4 (μg/mL)	HCl salt (μg/mL)	Fold increase	
			Free form	HCl salt
MP2	0.070			
AJ-037	103	5,000	×1,471	×71,428
AJ-039	2.98	25.2	×43	×360
AJ-206	102	>10,000	×1,457	>×140,000

### MIC values and intracellular activities of thiopeptide derivatives

To confirm the susceptibility of thiopeptide derivatives, the MIC values of MP2, AJ-037, AJ-039, and AJ-206 were tested. The MIC values were determined by resazurin microtiter assay (REMA), and CLR was compared as a control drug. The MIC values of all tested drugs against each MAC strain are presented in Table 2. Notably, all thiopeptide derivatives, including MP2, exhibited superior activity against both *M. avium* and *M. intracellulare* compared to the control drug CLR, as evidenced by the low MIC values and more effective inhibition of bacterial growth.

**TABLE 2:** MICs of antimycobacterial drugs against *Mycobacterium* strains^*b*^

		MIC (μg/mL)				
No.	Strains	CLR	MP2	AJ-037	AJ-039	AJ-206
1	*M. avium* (ATCC 700898)^*a*^	0.5	0.125	0.25	0.125	0.5
2	*M. avium* (CP000479.1)^*a*^	0.5	0.125	0.25	0.125	0.5
3	*M. avium* (SMC#6)	0.125	0.125	0.125	0.125	0.125
4	*M. avium* (SMC#7)	0.5	0.125	0.25	0.125	0.5
5	*M. avium* (SMC#1637)	0.125	0.125	0.125	0.125	0.125
6	*M. intracellulare* (ATCC 13950)^*a*^	0.25	0.125	0.25	0.125	0.5
7	*M. intracellulare* (SMC#7)	0.125	0.125	0.125	0.125	0.25
8	*M. intracellulare* (SMC#8)	0.25	0.125	0.25	0.125	0.5
9	*M. intracellulare* (SMC#9)	0.125	0.125	0.125	0.125	0.125
10	*M. intracellulare* (SMC#12)	0.25	0.125	0.25	0.125	0.5
11	*M. intracellulare* (SMC#699)	0.125	0.125	0.25	0.125	0.5
12	*M. intracellulare* (SMC#831)	0.125	0.125	0.125	0.125	0.125
13	*M. intracellulare* (SMC#2087)	0.125	0.125	0.125	0.125	0.125

^*a*^ Three reference strains were tested as controls.

^*b*^ The MIC breakpoints for the MAC were recommended by the Clinical and Laboratory Standards Institute. The resistant breakpoint for CLR was ≥32 µg/mL.

**Fig 2:**
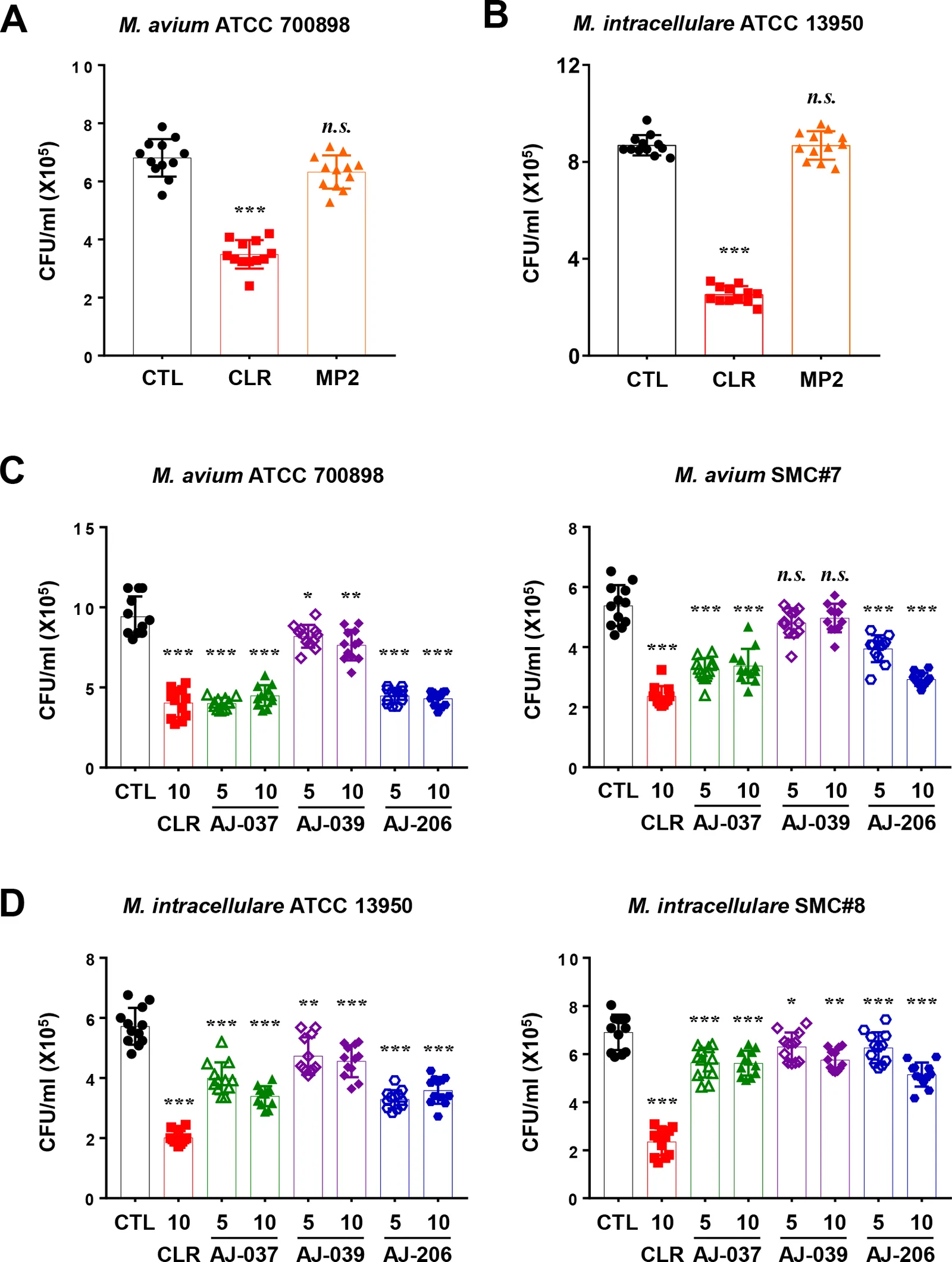
Evaluation of intracellular anti-MAC activities of thiopeptide derivatives in primarily cultured macrophages. BMDMs were infected with (**A**) *M. avium* ATCC 700898 or (**B**) *M. intracellulare* ATCC 13950 and treated with a 10 µg/mL dose of CLR and MP2. BMDMs were infected with (**C**) *M. avium* ATCC 700898 and *M. avium* SMC#7 or (**D**) *M. intracellulare* ATCC 13950 and *M. intracellulare* SMC#8. The cells were treated with 10 µg/mL CLR and the indicated doses (μg/mL) of thiopeptide derivatives. CLR treatment was used as a control. All experiments investigating the intracellular activities of drugs were assessed by plating serially diluted cell lysates on 7H10-OADC agar plates 72 h post-infection. The results are presented as the mean ± SD, and the Mann-Whitney test was used to evaluate significance. *P < 0.05; **P < 0.01; ***P < 0.001 vs CTL. *n.s*., not significant; CTL, untreated control.

After observing the inhibitory potential of thiopeptide derivatives against MAC, we evaluated the intracellular activities in MAC-infected murine bone marrow-derived macrophages (BMDMs) to determine whether each drug inhibited the intracellular growth of MAC. Cell viability was decreased at 20 µg/mL of MP2 and AJ-037, so 10 µg/mL was set as the maximum drug concentration (Fig. S4 in the supplemental material). Next, BMDMs were infected with *M. avium* ATCC 700898 and *M. intracellulare* ATCC 13950 and treated with CLR (10 µg/mL) and MP2 (10 µg/mL) for 72 hr (Fig. 2A and B). Interestingly, MP2, which is the backbone of the thiopeptide derivatives, was ineffective against *M. avium* ATCC 700898 and *M. intracellulare* ATCC 13950 in BMDMs, although a low MIC (0.125 µg/mL) value was observed. Subsequently, AJ-037, AJ-039, and AJ-206 were administered at two concentrations (5 µg/mL and 10 µg/mL) to *M. avium* ATCC 700898 and *M. avium* SMC#7-infected BMDMs (Fig. 2C). The results showed that the three compounds significantly inhibited intracellular bacterial growth compared to the untreated control (CTL) group. Similarly, the growth of *M. intracellulare* ATCC 13950 and *M. intracellulare* SMC#8 was also inhibited by thiopeptide derivatives (Fig. 2D). Notably, although all compounds were active against MAC, AJ-037 and AJ-206 remarkably inhibited the intracellular growth of MAC to a similar degree as CLR.

### Measurement of synergy with CLR

**Fig 3:**
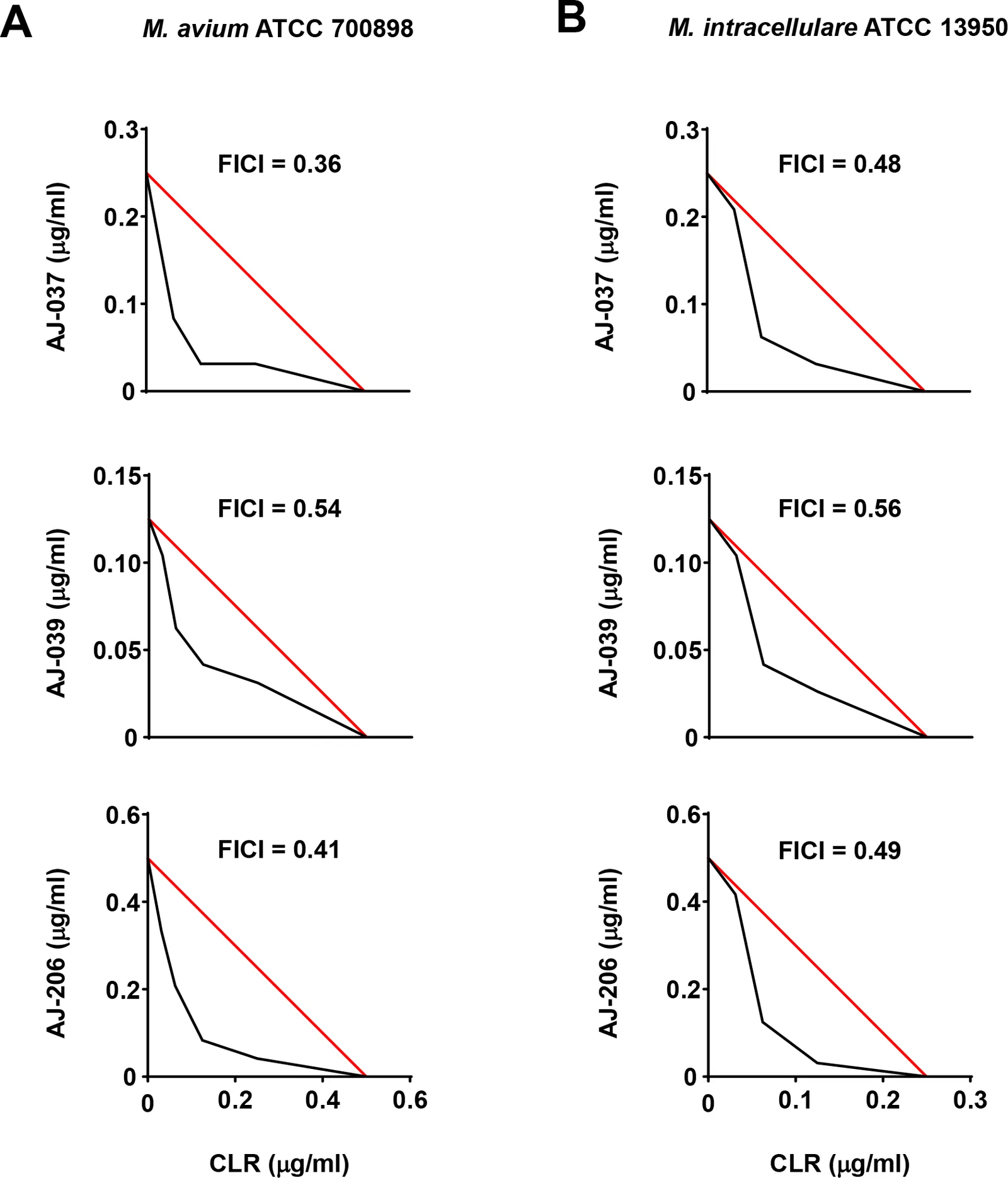
Isobologram analysis of the checkerboard assay with CLR and thiopeptide derivatives against MAC strains. Isobolograms of the combination of CLR and thiopeptide derivatives were created using a checkerboard assay against (**A**) *M. avium* ATCC 700898 or (**B**) *M. intracellulare* ATCC 13950. AJ-037, AJ-039, and AJ-206 were combined with CLR. The red line indicates reference MICs of a single drug. The black line indicates the result from the checkerboard assay. The black line below the red line indicates the synergistic effect of the two drugs. Synergistic effects were observed when CLR interacted with AJ-037 or AJ-206, and an additive effect was shown between CLR and AJ-039 in all tested MACs. The data are expressed as the average of three independent experiments.

CLR is a cornerstone in the treatment of MAC-infected patients, but CLR monotherapy is strictly prohibited because it can induce resistance (13, 37). Therefore, to identify whether thiopeptide derivatives have synergistic effects in combination with CLR, we first conducted a checkerboard assay. The drug-drug interactions were calculated using a fractional inhibitory concentration index (FICI) for each combination. The results against *M. avium* ATCC 700898 and *M. intracellulare* ATCC 13950 are presented as isobolograms (Fig. 3). The black line was drawn below the red line, indicating the synergistic effect of CLR and the thiopeptide derivative. Synergy was defined as an FICI of 0.5 or less, as previously reported (38 – 40). Surprisingly, the combination of CLR with AJ-037 and AJ-206 showed the strongest synergistic effect against *M. avium* ATCC 700898, with FICI values of 0.36 and 0.41, respectively. Additionally, in *M. intracellulare* ATCC 13950, the FICI values of AJ-037 and AJ-206 were 0.48 and 0.49, indicating a relatively weak but synergistic effect with CLR. However, AJ-039 combined with CLR had an additive effect, with FICI values of 0.54 (*M. avium* ATCC 700898) and 0.56 (*M. intracellulare* ATCC 13950). As previously reported, synergistic effects were not observed in the combination of CLR with moxifloxacin (MXF) and bedaquiline (BDQ), so the two drugs were used as controls (Fig. S5 in the supplemental material) (41, 42).

**Fig 4:**
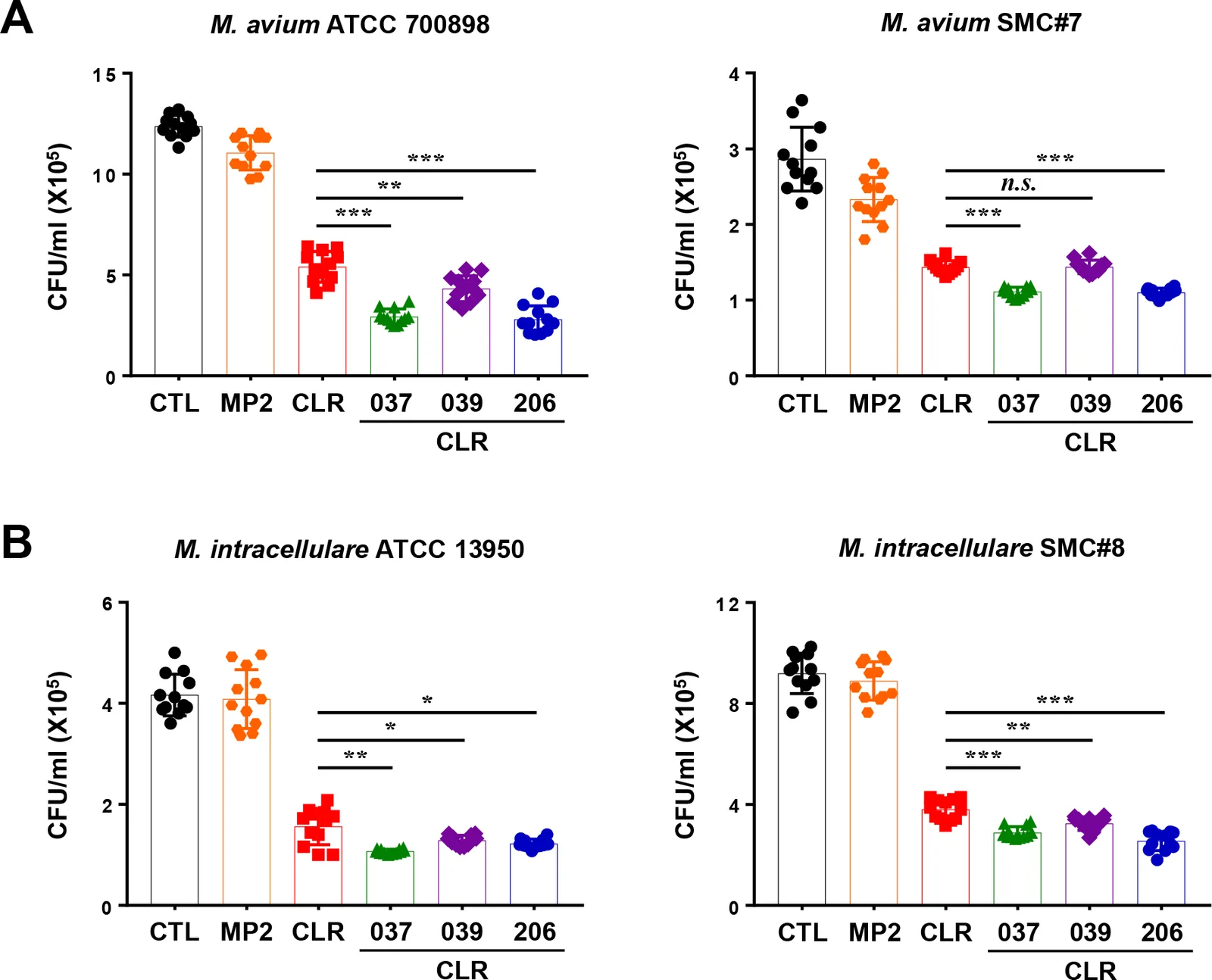
Evaluation of the intracellular anti-MAC activities of the CLR and thiopeptide derivative combinations in macrophages. Intracellular activities of drug combinations were performed against a variety of MAC strains. BMDMs were infected with (**A**) *M. avium* ATCC 700898 and *M. avium* SMC#7 or (**B**) *M. intracellulare* ATCC 13950 and *M. intracellulare* SMC#8. CLR (10 µg/mL) was combined with AJ-037 (10 µg/mL), AJ-039 (10 µg/mL), and AJ-206 (10 µg/mL). MP2 (10 µg/mL) and CLR (10 µg/mL) were used as controls. All experiments investigating the intracellular activities of drugs were assessed by plating serially diluted cell lysates on 7H10-OADC agar plates at 72 h post-infection. The results are presented as the mean ± SD, and the Mann-Whitney test was used to evaluate significance. *P < 0.05; **P < 0.01; ***P < 0.001 vs CTL. *n.s*., not significant; CTL, untreated control.

Next, we investigated the intracellular anti-MAC activity in BMDMs to confirm whether the synergistic effect could improve their ability to kill MAC. The concentration of all drugs was fixed at 10 µg/mL, and the intracellular activities of a single CLR versus the combination were compared. As shown in Fig. 4, the combination of thiopeptide derivatives and CLR showed synergistic inhibition of bacterial growth inside macrophages in the majority of MAC strains. These results were consistent with the results obtained from the checkerboard assay. Exceptionally, the activity of AJ-039 combined with CLR against *M. avium* SMC#7 was not significant compared to that of CLR alone, which correlated with the activity of AJ-039 alone, as shown in Fig. 2C. Collectively, our data clearly suggested that AJ-037 and AJ-206 specifically potentiate macrolide activity.

### Activities of thiopeptide derivatives against macrolide-resistant MAC strains

We also evaluated thiopeptide derivatives against macrolide-resistant MAC strains to evaluate their use in the treatment of patients with macrolide-resistant MAC. All macrolide-resistant strains tested in this study had a point mutation at position 2058 or 2059 of the 23S rRNA gene. The MIC results against macrolide-resistant MAC strains are summarized in Table 3. Interestingly, thiopeptide derivatives showed inhibitory activity against macrolide-resistant MAC strains as well as spotting growth assays (Fig. S6 in the supplemental material).

**TABLE 3:** MICs of antimycobacterial drugs against macrolide-resistant Mycobacterium strains^*a*^

No.	Strains	23 s rRNA mutation A2058/A2059	MIC (μg/mL)			
			CLR	AJ-037	AJ-039	AJ-206
1	*M. avium* (SMC#417)	C/A	>64	0.125	0.125	0.125
2	*M. avium* (SMC#420)	C/A	>64	0.5	0.5	0.5
3	*M. avium* (SMC#422)	A/G	>64	1	4	1
4	*M. avium* (SMC#1213)	T/A	>64	0.5	2	1
5	*M. avium* (SMC#1216)	C/A	>64	0.125	0.125	0.125
6	*M. intracellulare* (SMC#400)	C/A	>64	0.25	0.125	0.5
7	*M. intracellulare* (SMC#414)	A/G	>64	0.125	0.125	0.125
8	*M. intracellulare* (SMC#418)	G/A	>64	0.25	0.125	0.5
9	*M. intracellulare* (SMC#104)	G/A	>64	0.125	0.125	0.125
10	*M. intracellulare* (AMC#24)	G/A	>64	0.125	0.125	0.125

^*a*^ The MIC breakpoints for the MAC were recommended by the Clinical and Laboratory Standards Institute. The resistant breakpoint of CLR was ≥32 μg/mL.

**Fig 5:**
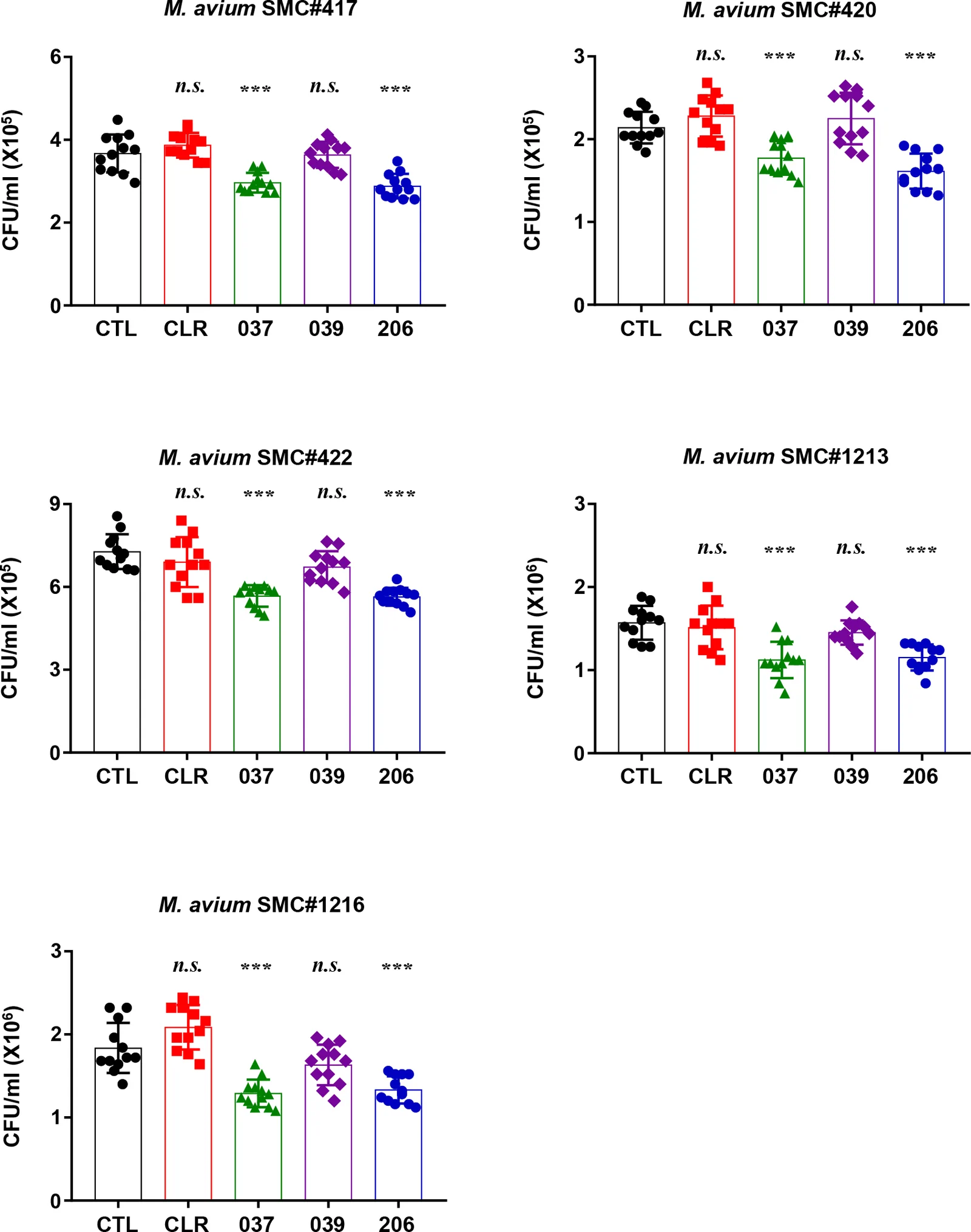
Assessment of intracellular activities of thiopeptide derivatives against various macrolide-resistant *M. avium* clinical isolates. Intracellular anti-MAC activities of thiopeptide derivatives were tested against macrolide-resistant *M. avium* clinical isolates. BMDMs were infected with five macrolide-resistant *M. avium* clinical isolates. They were treated with 10 µg/mL CLR and 10 µg/mL thiopeptide derivatives. CLR treatment was used as a control. All experiments investigating the intracellular activities of drugs were assessed by plating serially diluted cell lysates on 7H10-OADC agar plates at 72 h post-infection. The results are presented as the mean ± SD, and the Mann-Whitney test was used to evaluate significance. *P < 0.05; **P < 0.01; ***P < 0.001 vs CLR. *n.s*., not significant; CTL, untreated control.

**Fig 6:**
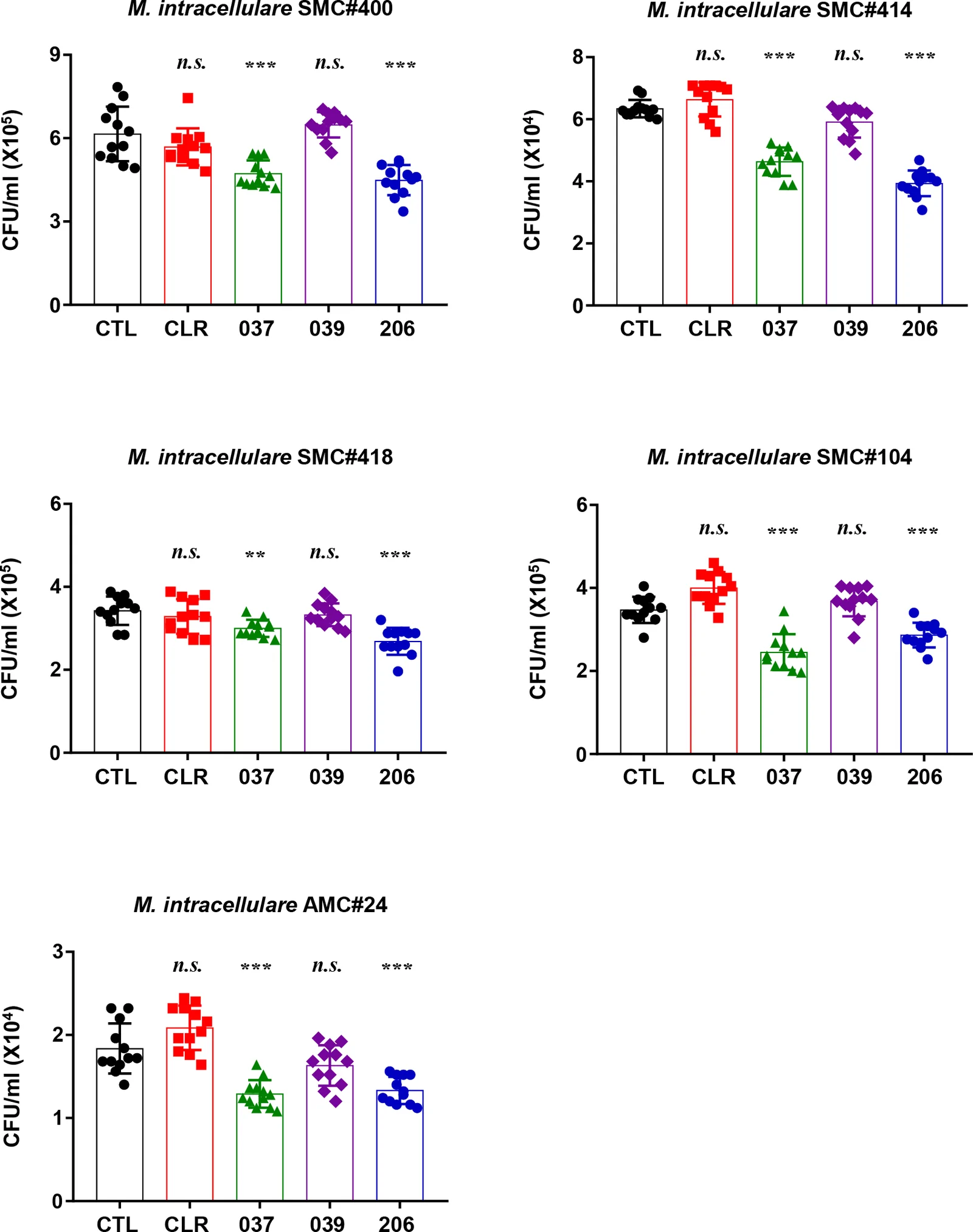
Assessment of intracellular activities of thiopeptide derivatives against various macrolide-resistant *M. intracellulare* clinical isolates. Intracellular anti-MAC activities of thiopeptide derivatives were tested against macrolide-resistant *M. intracellulare* clinical isolates. BMDMs were infected with five macrolide-resistant *M. intracellulare* clinical isolates. They were treated with 10 µg/mL CLR and 10 µg/mL thiopeptide derivatives. CLR treatment was used as a control. All experiments investigating the intracellular activities of drugs in BMDMs were assessed by plating serially diluted cell lysates on 7H10-OADC agar plates at 72 h post-infection. The results are presented as the mean ± SD, and the Mann-Whitney test was used to evaluate significance. *P < 0.05; **P < 0.01; ***P < 0.001 vs CLR. *n.s*., not significant; CTL, untreated control.

Next, we evaluated the intracellular activities of thiopeptide derivatives against macrolide-resistant MAC strains. All strains tested in the MIC assay were assessed inside BMDMs. Five *M. avium* clinical isolates were infected and treated with 10 µg/mL CLR and thiopeptide derivatives (Fig. 5). There was CLR resistance in macrolide-resistant MAC strains. AJ-037 and AJ-206 notably inhibited intracellular bacterial growth. In the same manner, thiopeptide derivatives were effective against macrolide-resistant *M. intracellulare* clinical isolates (Fig. 6). These results strongly suggest that thiopeptide derivatives may be effective antimicrobial antibiotics for macrolide-resistant MAC strains. This indicated that these derivatives could be a promising solution to the problem of macrolide-resistant MAC infections.

## DISCUSSION

In this study, the *in vitro* drug susceptibility and intracellular antimycobacterial activities of AJ-037, AJ-039, and AJ-206, which were newly designed based on the thiopeptide MP2, were evaluated in comparison with CLR, which is a primary drug for the treatment of MAC-PD. Thiopeptide derivatives have shown potent activities against MAC, including macrolide-resistant bacteria, and have the potential to be effective anti-MAC drugs. MP2 exhibits a low *in vitro* MIC (0.125–0.5 µg/mL) for the tested MAC strains, but its intracellular activity is completely abrogated in MAC-infected BMDMs (Table 2; Fig. 2A and B). A possible explanation of this waning activity of MP2 may result from an unsuitable solubility profile that prevents it from entering the cell membrane to exert its antibacterial activity. Therefore, poorly soluble MP2 has limitations in targeting intracellular pathogens. Synthetic efforts to increase the solubility of MP2 have led us to develop several novel compounds, such as AJ-037, AJ-039, and AJ-206. In particular, AJ-037 and AJ-206 significantly reduced the number of MAC CFUs inside macrophages (Fig. 2C and D).

In drug development for the treatment of MAC-PD, one of the major hurdles is that the activities of most drugs, except for macrolides, differ between *in vitro* and *in vivo* studies (43 – 45). For example, rifampicin, one of the drugs in the standard treatment regimen, showed little intracellular activity against MAC despite its extremely low *in vitro* MICs for unknown reasons (46). Thus, this discrepancy between the *in vitro* MIC and the intracellular activity of a certain drug makes it difficult to find promising anti-MAC drugs. In this regard, thiopeptide derivatives demonstrated promising anti-MAC potential based on *in vitro* susceptibility profiles and intracellular activities comparable to CLR. They had antimicrobial activity with very low MICs and presented excellent inhibitory activities against most MAC clinical strains. Therefore, the *in vivo* efficacy evaluation of thiopeptide derivatives in proper animal models will further warrant their development for clinical use.

Additionally, companion drugs such as EMB and RIF are only empirically used to prevent the development of macrolide resistance, and their anti-MAC activities have not been confirmed until now (47 – 50). Along with the effort to develop new agents for MAC infections, there have been many attempts to find the best partner for CLR to improve treatment outcomes (50, 51). For example, our previous study reported that a clofazimine (CFZ)-containing regimen instead of RIF of the standard regimen significantly reduced bacterial burdens in all organs and reduced the treatment duration (46). Thus, the replacement of RIF with CFZ in the treatment regimen resulted in more favorable outcomes, and a synergistic effect of CLR with CFZ was demonstrated. In the present study, the checkerboard assay was carried out to confirm the synergistic effect of the combinations between individual thiopeptide derivatives and CLR against MAC. The findings revealed that two compounds, AJ-037 and AJ-206, had FICI values of less than 0.5 when combined with CLR, indicating their evident synergistic effects with CLR (Fig. 3). In addition, the combination of CLR and thiopeptide derivatives significantly reduced MAC numbers compared to CLR alone in macrophages (Fig. 4). However, changes in CFUs of *M. avium* SMC#7 when given the combination of CLR and AJ-039 were not significant, which aligns with the fact that AJ-039 alone did not reduce CFUs in Fig. 2C. Since the new thiopeptide derivatives developed in this study clearly potentiated CLR activity *in vitro* and intracellularly against MAC, further optimization of these compounds is worthy of attention.

When used in combination with other anti-MAC drugs, it should be evaluated whether thiopeptide derivatives induce cross-resistance to macrolides because these derivatives target bacterial ribosomes. The mechanisms of macrolide resistance in MAC have been studied at the molecular level, and point mutations in the 23S rRNA gene at positions 2058 or 2059 are well known to be responsible for macrolide resistance in clinical isolates (52, 53). This study included macrolide-resistant MAC clinical strains to identify whether they have cross-resistance. Thiopeptide derivatives exhibited outstanding inhibitory activity against the majority of macrolide-resistant MAC clinical isolates (Table 3). Eight out of the 10 strains tested had MICs ≤ 0.5 µg/mL. However, these effects may occur with any drug with a different, albeit less interactive, mechanism of action. Therefore, we confirmed the presence or absence of cross-resistance once again by confirming the intracellular activity against macrolide-resistant MAC clinical isolates. AJ-037 and AJ-206 treatment led to a significant decrease in CFUs in BMDMs infected with macrolide-resistant MAC clinical isolates while still exhibiting antimicrobial activity (Fig. 5 and 6). However, AJ-039 had relatively little activity in the macrophage infection model compared to its low MIC activity, which is likely because its aqueous solubility did not increase significantly compared to MP2, the derivative backbone. In addition, a few macrolide-resistant *M. avium* strains showed relatively high MICs, but the intracellular activities had the same significance as other strains. Our results highlight that there is no cross-resistance between thiopeptide derivatives and CLR, even though they both target bacterial ribosomes. It has been reported in other species that resistance to thiopeptide is associated with mutations in H43 and H44 of the 23S rRNA or in *rplk*, a gene encoding ribosomal protein L11 (54 – 56). Moreover, thiopeptides are active against many antibiotic-resistant bacteria, such as MRSA, vancomycin-resistant enterococci, and penicillin-resistant *Streptococcus pneumonia* (16, 24). The novel target of thiopeptides, different from those of macrolides, supports our results that they have no cross-resistance. Therefore, thiopeptide derivatives are promising antibiotics for the treatment of macrolide-resistant MAC, for which there is no alternative to date.

Our study has several limitations. First, a formulation study for *in vivo* lung delivery should be conducted. Compared to CLR, thiopeptide derivatives have twice as large molecular weights. The delivery limitation imposed by these properties can be overcome by developing a liposome-based formulation of the compounds. Second, in this study, only synergy with macrolides was confirmed. Since a multidrug regimen is used in the treatment of MAC infection, it is necessary to verify the synergy and cross-resistance with other anti-MAC drugs, such as EMB and RIF. Third, the exact mechanism of resistance to thiopeptide derivatives in MAC has not been elucidated. To effectively tackle the issue of resistance that may arise during future clinical use, it will be essential to generate resistant mutants and clarify the precise mechanisms underlying resistance through their study. Additionally, further study is needed to determine whether combinations with our compounds can reduce the required dose of macrolide.

Nevertheless, our findings suggest that thiopeptide derivatives are highly effective candidates for addition to the current anti-MAC antibiotic regimen. The results of AJ-037 and AJ-206 testing, which were performed in this study, proved that they can be used as anti-MAC antibiotics together with CLR; furthermore, they are also effective in the treatment of macrolide-resistant MAC strains. This study is advantageous in that it evaluated the MICs, intracellular activity, and synergistic effects of antibiotics, including in macrolide-resistant strains. Ultimately, we concluded that the thiopeptide derivatives AJ-037 and AJ-206 were highly effective candidates as alternatives to current anti-MAC antibiotics. Notwithstanding, further research including animal models is needed to support the continued development of thiopeptide derivatives.

## MATERIALS AND METHODS

### Analytical methods

^1^H and ^13^C NMR experiments were recorded on a Bruker AV VIII 400 spectrometer (Billerica, MA, USA) or a Bruker Avance III 600 spectrometer (Billerica, MA, USA). Chemical shifts (δ scale) are reported in parts per million (ppm) relative to the central peak of the solvent. Coupling constants (J) are given in hertz (Hz). Spectra were obtained in the following solvents (reference peaks included for ^1^H and ^13^C NMR): CD_3_OD (^1^H NMR: 3.35 ppm. 4.78 ppm; ^13^C NMR: 49.3 ppm), dimethyl sulfoxide (DMSO)-*d6* (^1^H NMR: 2.50 ppm; ^13^C NMR: 39.5 ppm). NMR experiments were performed at room temperature. Chemical shift values for all ^1^H and ^13^C spectra are reported in ppm. ^1^H NMR multiplicities are reported as s = singlet, d = doublet, t = triplet, q = quartet, m = multiplet, and br = broad. HRMS were obtained at the Korea Basic Science Institute (Daegu, South Korea) on a Jeol JMS 700 high resolution (Tokyo, Japan) using a fast atom bombardment ionization source or at Gyeongsang National University (Jinju, South Korea) on a Waters Xevo G2-Xs high-resolution mass spectrometer (Milford, MA, USA) using electrospray ionization source. The purity of the final compounds was determined by a Shimadzu 20A HPLC (Kyoto, Japan) system equipped with a YMC-Triart C18 column (5 µm, 150 × 3.0 mm, 40°C, UV: 254 nm). The gradient was used with 5% acetonitrile in water (containing 0.1% trifluoroacetic acid) to 100% acetonitrile, and the flow rate was 1.0 mL/min.

### Aqueous kinetic solubility

The aqueous kinetic solubility was determined by preparing a 20 mM DMSO stock solution of test compounds and incubating an aliquot of this solution in deionized water at pH 7.4 to set a 1% final concentration of DMSO. In the case of HCl salt compounds, 1–2 mg of compound was weighed and dissolved in deionized water to yield an approximately 10 mg/mL solution. After initial mixing using brief vortexing and sonication, samples were equilibrated by shaking for 2 h at room temperature. Suspensions or solutions with visible particles were filtered through 0.22 µm PVDF membrane filters. The dissolved drug concentration was analyzed using UPLC-MS. To determine the solubility of the HCl salt, 1 mg of compound was weighed into a 2 mL glass tube, and a fixed volume of deionized water was added to yield an approximately 10 mg/mL solution. The solution was equilibrated by shaking for 24 h at room temperature. After equilibration, the vials were visually examined. If a clear solution was obtained, solubility was reported as >mg/mL.

### MAC strains and bacterial culture conditions

Reference strains were purchased from the American Type Culture Collection (ATCC; Manassas, VA, USA). All clinical MAC isolates, including macrolide-susceptible and macrolide-resistant strains, were kindly provided by Samsung Medical Center (SMC; Seoul, South Korea) and Asan Medical Center (AMC; Seoul, South Korea). All strains were cultivated in Middlebrook 7H9 liquid broth (Difco Laboratories, Detroit, MI) supplemented with 10% oleic acid-albumin-dextrose-catalase (OADC) or plated on Middlebrook 7H10 agar (Difco Laboratories) with 10% OADC enrichment at 37°C.

### Antimicrobial agents

CLR was purchased from Tokyo Chemical Industry (TCI; Tokyo, Japan), and thiopeptide derivatives were provided by A&J Science Co., Ltd. (Daegu, South Korea). All reagents were reconstituted in DMSO. They were diluted in Dulbecco’s phosphate-buffered saline (DPBS; Biowest, Nuaillé, France) after complete dissolution for the *in vitro* study.

### *In vitro* susceptibility testing

The MICs of all the reagents were determined using the REMA method based on guidelines by the Clinical and Laboratory Standards Institute (57). For the MIC test, bacteria were grown in 7H9 broth supplemented with 10% OADC at 37°C until midlog phage, i.e., OD600 = 0.3–0.5. The cultures were diluted to an OD600 = 0.001 in 7H9 broth with 5% OADC. In a 96-well plate, the drugs were serially diluted twofold in 100 mL of 7H9 broth with 5% OADC, and 100 µL of bacterial suspension was added to the following final drug concentrations: 0.125–64 μg/mL. Two controls (broth only and bacteria only) were incubated to check for other contamination (broth only) and the growth of tested bacteria (bacteria only). Following incubation at 37°C for 5–7 d, MIC values were deduced by reading the minimum concentration of drugs by REMA. All MIC results were validated by independently repeating the test at least three times.

### Murine BMDM culture

BMDMs were obtained from 6-week-old female BALB/c mice. Bone marrow cells were differentiated into BMDMs using Dulbecco’s Modified Eagle Medium (Biowest) supplemented with 10% fetal bovine serum (Biowest) and 10% L929 cell supernatant as previously described (58).

### Cell cytotoxicity test

Cell viability was evaluated using trypan blue cell counting. BMDMs were plated at 4 × 10^5^ cells/mL along with antibiotics in a 48-well cell culture plate. The antibiotics were serially diluted twofold from the highest concentration of 20 μg/mL to 1.25 μg/mL. After overnight incubation at 37°C with 5% CO_2_, BMDMs were isolated, and viable cells were counted using trypan blue dye exclusion. Cell viability was calculated as a percentage compared to the control. All experiments were repeated at least three times independently.

### Intracellular anti-MAC activity

BMDMs were seeded at 4 × 10^5^ cells/mL in a 48-well plate and incubated overnight at 37°C. MAC strains were infected at a multiplicity of infection of 3 for 4 h and washed with DPBS twice. The cells were then cultured in the absence or presence of drugs in triplicate wells for 72 h. After incubation, the cells were lysed with 0.05% Triton X-100, and the cell lysates serially diluted with DPBS were spotted on 7H10 agar plates supplemented with OADC to count the number of viable bacteria. Colonies were enumerated after 7 d of incubation at 37°C, and the values are reported as the mean CFU ± standard deviation (SD) per mL. All experiments were repeated at least three times independently.

### Checkerboard assay

The checkerboard assay was used to assess the synergistic effects of two antimicrobial agents in combination (38 – 40). To evaluate the efficacy of the combination of agent A and agent B, a fractional inhibitory concentration (FIC) index was calculated as follows: FICI = (FIC of agent A) + (FIC of agent B) = (MIC of agent A in combination/MIC of agent A alone) + (MIC of agent B in combination/MIC of agent B alone). Synergism is defined as FICI ≤0.5, additive as 0.5 < FICI ≤ 1, indifferent as 1 < FICI ≤ 4, and antagonistic as FICI >4. The cultured bacteria were diluted to a 0.005 OD600 in 7H9 broth containing 5% OADC, and the antimicrobial agents were prepared by serial dilutions to a final concentration of 0.03–4 μg/mL. For the evaluation of inhibitory concentrations, the plates were incubated at 37°C for 5–7 d. Then, MIC values were assessed by REMA, and the FICI was calculated. All results for the tested MAC strains were verified by repeating at least three times independently.

### Genomic DNA extraction

Macrolide-resistant MAC clinical isolates were grown in 7H9 broth supplemented with OADC at 37°C until log phage. Mycobacterial cultures were collected by centrifugation, and the pellet was resuspended in 500 µL of TE buffer and 10 µL of 100 mg/mL lysozyme. After overnight incubation at 37°C, the bacterial lysate was incubated with 100 µL of 10% SDS and 15 µL of 20 mg/mL proteinase K at 65°C for 3 h. Then, the bacteria were treated with 5 M NaCl for 10 min, and 80 µL of CTAB/NaCl was added for 10 min at 65°C. DNA extraction was performed by adding 700 µL chloroform/isoamyl alcohol (24:1) and repeating twice. Isopropanol was added, the sample was incubated for at least 1 h at −20°C, and the pellet was washed out with 75% EtOH. Genomic DNA was completely dried, resuspended in sterile dH_2_O, and stored at −20°C until use.

### PCR amplification, sequencing of antibiotic resistance genes, and sequence analysis

PCR amplification and sequencing for CLR were based on 23S rRNA. The primers and PCR conditions were as reported previously (59). PCR was carried out in a final volume of 20 µL and contained EzPCR Basic 5 × premix (ELPIS, Daejeon, South Korea), 13 µL of sterile dH_2_O, 1 µL of each forward and reverse primer, and 1 µL of template DNA. The PCR products were run on 1% agarose gels and purified using a NEXprep Gel/PCR Purification Mini Kit (Geneslabs, Gyeonggi, South Korea) for DNA cleanup. Purified PCR products were sequenced by Sanger sequencing in Bionics (Seoul, South Korea) with their gene-specific forward and reverse primers. Sequences obtained from Bionics were subjected to BLAST searches to compare nucleotide sequences with reference strains.

### Spotting growth assay

In a 96-well plate, serial 10-fold dilutions of bacteria were prepared in 7H9 broth supplemented with OADC. Then, antibiotics at 1 × MIC of each strain were added together and incubated at 37°C. After 6 d, 5 µL of each dilution was spotted on 7H10 agar plates.

### Statistical analysis

All experiments were repeated at least three times, and consistent results were obtained. Statistical analysis was conducted using GraphPad Prism version 8 (San Diego, CA, USA). The Mann-Whitney test was used to assess differences between the two groups, and ANOVA corrected with Tukey’s test was used to evaluate differences between multiple groups. A *P*- value of < 0.05 was considered statistically significant.

## Data Availability

Sequence analysis was performed to identify a point mutation at position 2058 or 2059 of the 23s rRNA gene. Nucleotide replacements in macrolide-resistant *Mycobacterium* strains have been compiled in Table 3. All data of this article are available in the article and the supplemental material.

## SUPPLEMENTAL MATERIAL

Supplemental figures spectrum.01825-23-s0001.docx Structural characterizations, cell cytotoxicity, checkerboard assay, and spotting growth assay.

ASM does not own the copyrights to Supplemental Material that may be linked to, or accessed through, an article. The authors have granted ASM a non-exclusive, world-wide license to publish the Supplemental Material files. Please contact the corresponding author directly for reuse.
