# Supplementary material for: In vitro and intracellular activities of novel thiopeptide derivatives against macrolide-susceptible and macrolide-resistant Mycobacterium avium complex
Source: Microbiol Spectr. 2023 Aug 18;11(5):e01825-23. doi: 10.1128/spectrum.01825-23 (PMC10580953; doi:10.1128/spectrum.01825-23)
Supplement: Supplemental figures — Structural characterizations, cell cytotoxicity, checkerboard assay, and spotting growth assay. [file spectrum.01825-23-s0001.docx]

**Supplemental Materials**

*In vitro* and intracellular activities of novel thiopeptide derivatives against macrolide-susceptible and macrolide-resistant *Mycobacterium avium* complex

Jiyun Park^a†^, Lee-Han Kim^a†^, Ju Mi Lee^a^, Sangwon Choi^a^, Young-Jin Son^b^, Hee-Jong Hwang^b*^, Sung Jae Shin^a*^

^a^Department of Microbiology, Institute for Immunology and Immunological Disease, Brain Korea 21 Project for Graduate School of Medical Science, Yonsei University College of Medicine, Seoul 03722, South Korea

^b^A&J Science Co., Ltd., 80 Chumbok Ro, Dong Gu, Daegu 41061, South Korea

**SUPPLEMENTAL FIGURES**

^1^H NMR
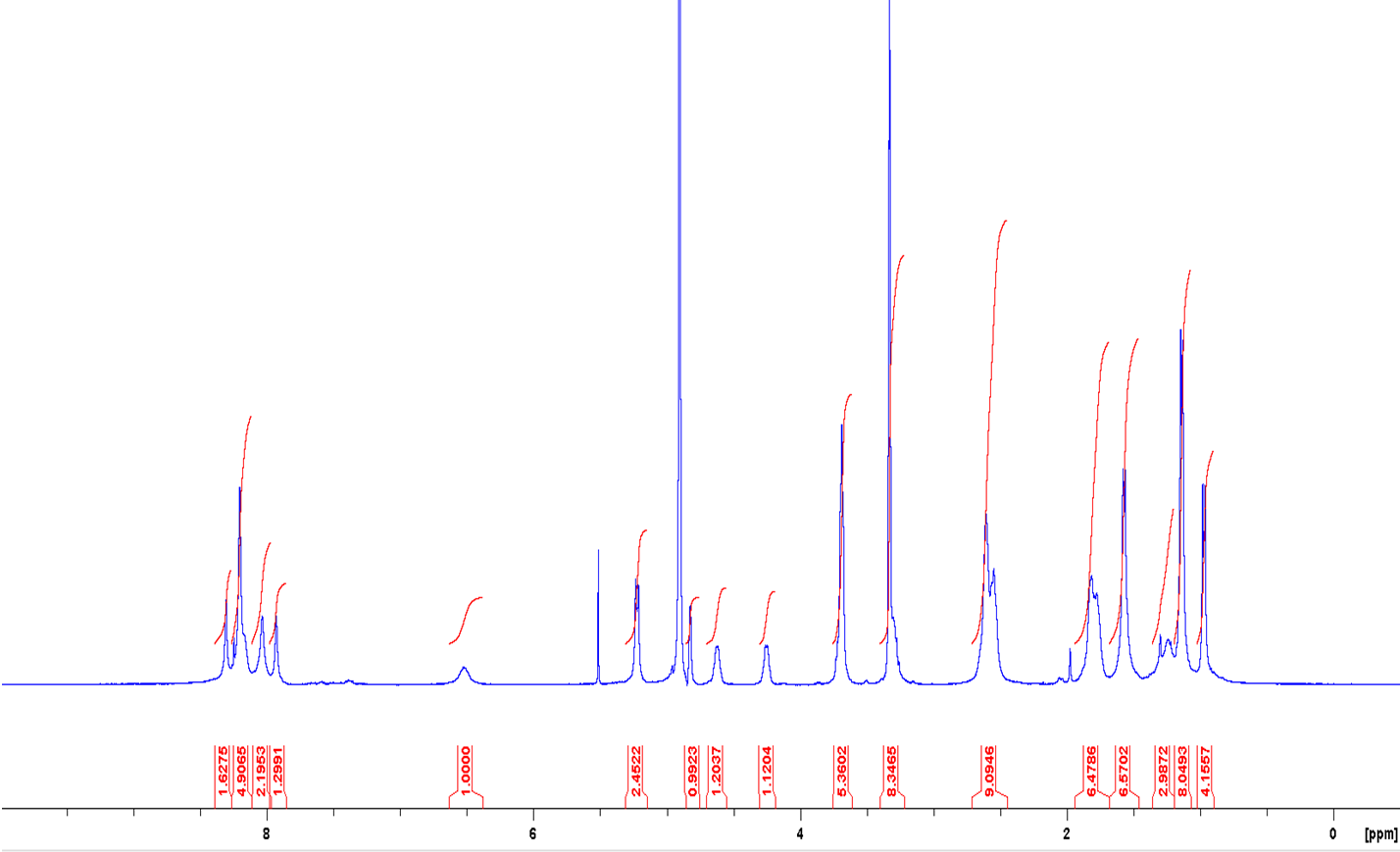

^13^C NMR
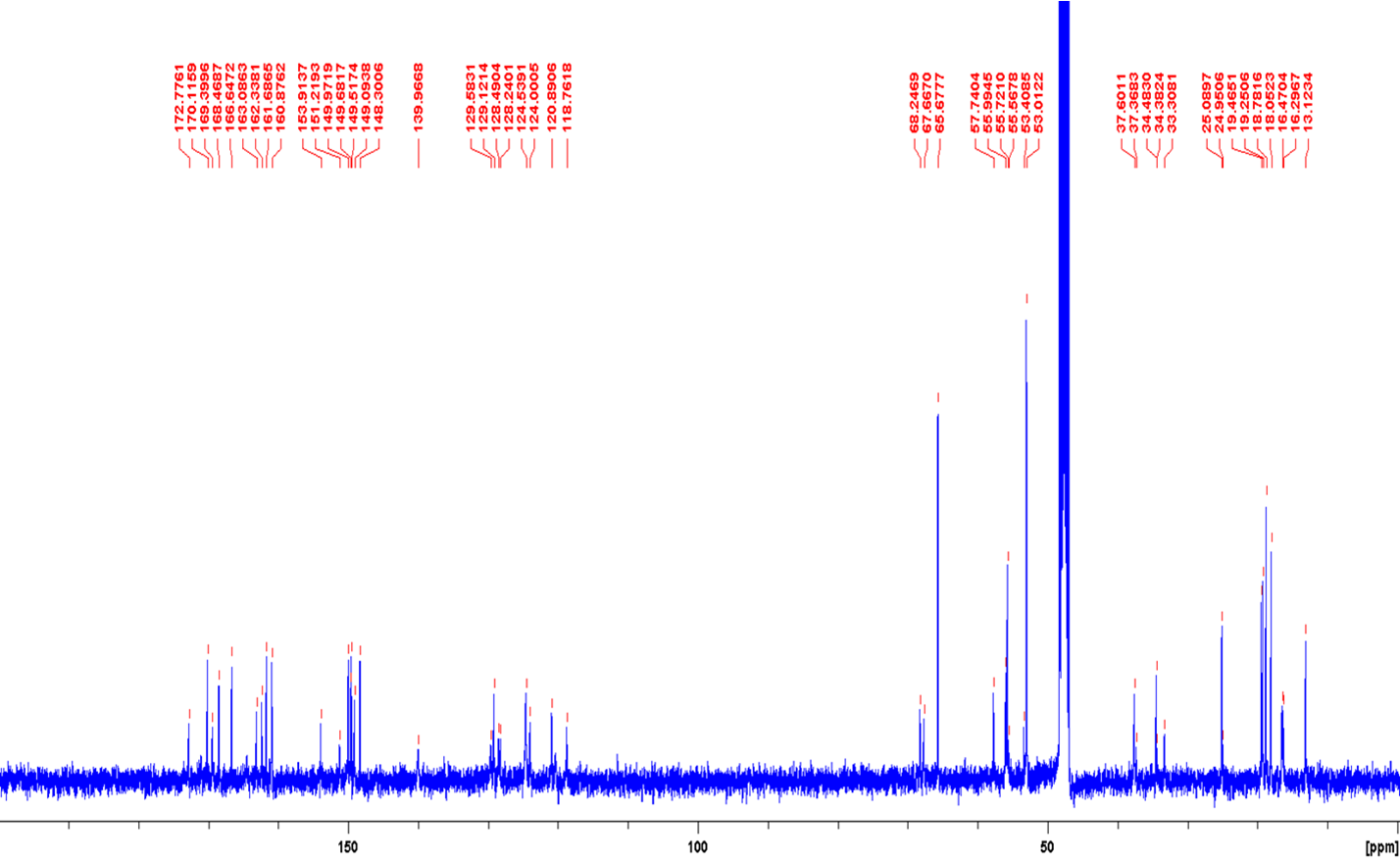


HPLC


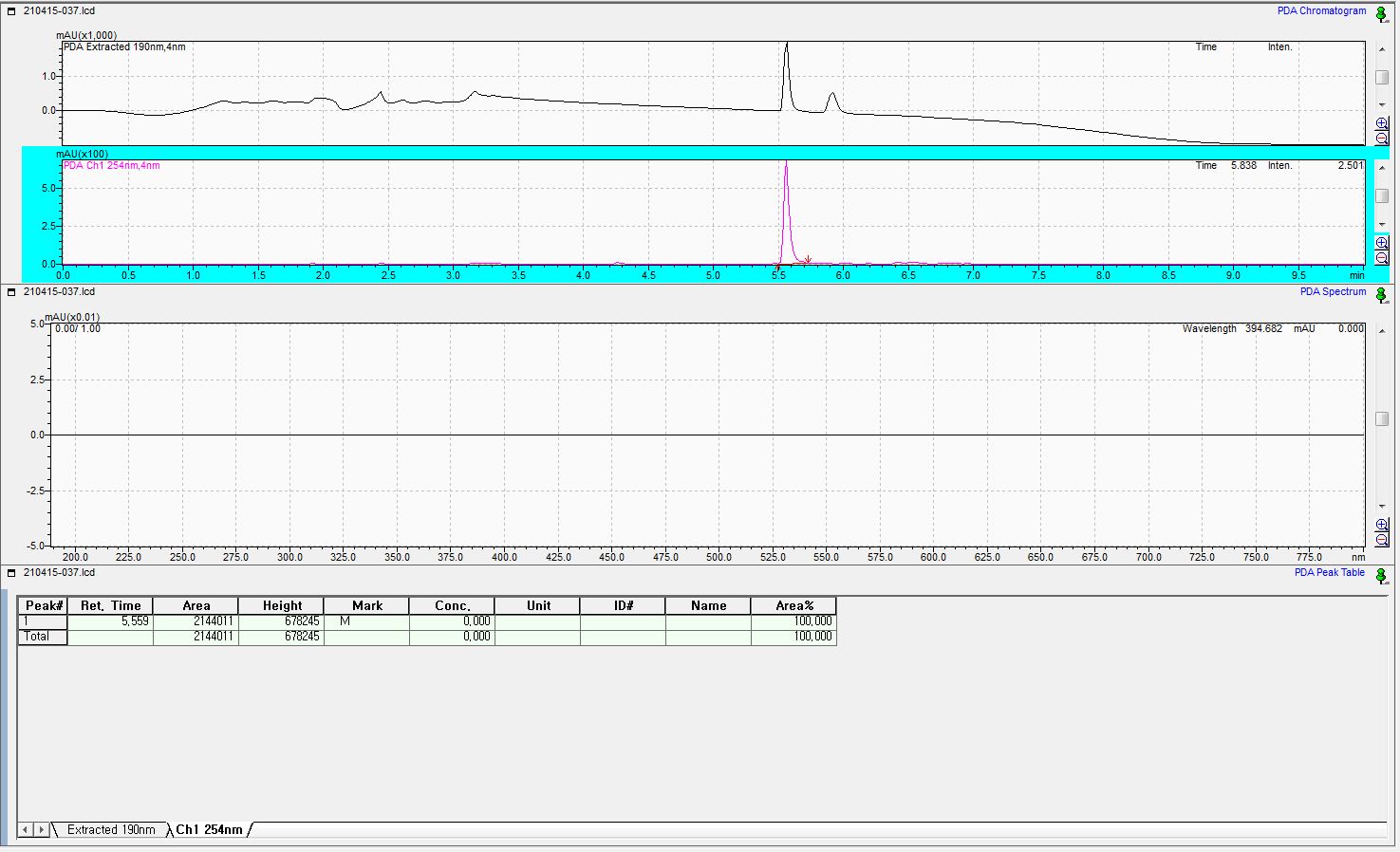


**Fig S1. ^1^H NMR, ^13^C NMR, HPLC and HRMS data of AJ-037**

**^1^H NMR (400 MHz, CD_3_OD):** δ 8.31 (s, 1H), 8.22 (d, *J* = 17.2 Hz, 4H), 8.03 (s, 2H), 7.93 (s, 1H), 6.52 (bs, 1H), 5.22 (d, *J* = 8.4 Hz, 2H), 4.82 (s, 1H), 4.63 - 4.60 (m, 1H), 4.25 – 4.23 (m, 1H), 3.69 (broad band, 5H), 2.71 – 2.39 (m, 9H), 1.88 – 1.64 (m, 4H), 1.57 (d, *J* = 6.0 Hz, 6H), 1.34 – 1.18 (m, 2H), 1.14 (d, *J* = 6.1 Hz, 5H), 0.97 (d, *J* = 6.5 Hz, 3H).

**^13^C NMR (101 MHz, CD_3_OD):** δ 172.8, 170.1, 169.4, 168.5, 166.6, 163.1, 162.3, 161.7, 160.9, 153.9, 151.2, 150.0, 149.7, 149.5, 149.1, 148.3, 140.0, 129.6, 129.1, 128.5, 128.2, 124.5, 124.0, 120.9, 118.8, 68.2, 67.7, 65.7, 57.7, 56.0, 55.7, 55.6, 53.4, 53.0, 37.6, 37.4, 34.5, 34.4, 33.3, 25.1, 25.0, 19.5, 19.2, 18.8, 18.0, 16.5, 16.3, 13.1.

**HRMS (FAB):** *m/z* calc for [C_52_H_56_N_14_O_9_S_6_+H]^+^ 1213.2752, found 1213.2759.

**HPLC**: flow rate 1.0 mL / min, t_R_ (5% -> 100 % acetonitrile / 95% -> 0% water (containing 0.1% TFA)) = 5.56 min, area 2144011, purity >99%.

^1^H NMR


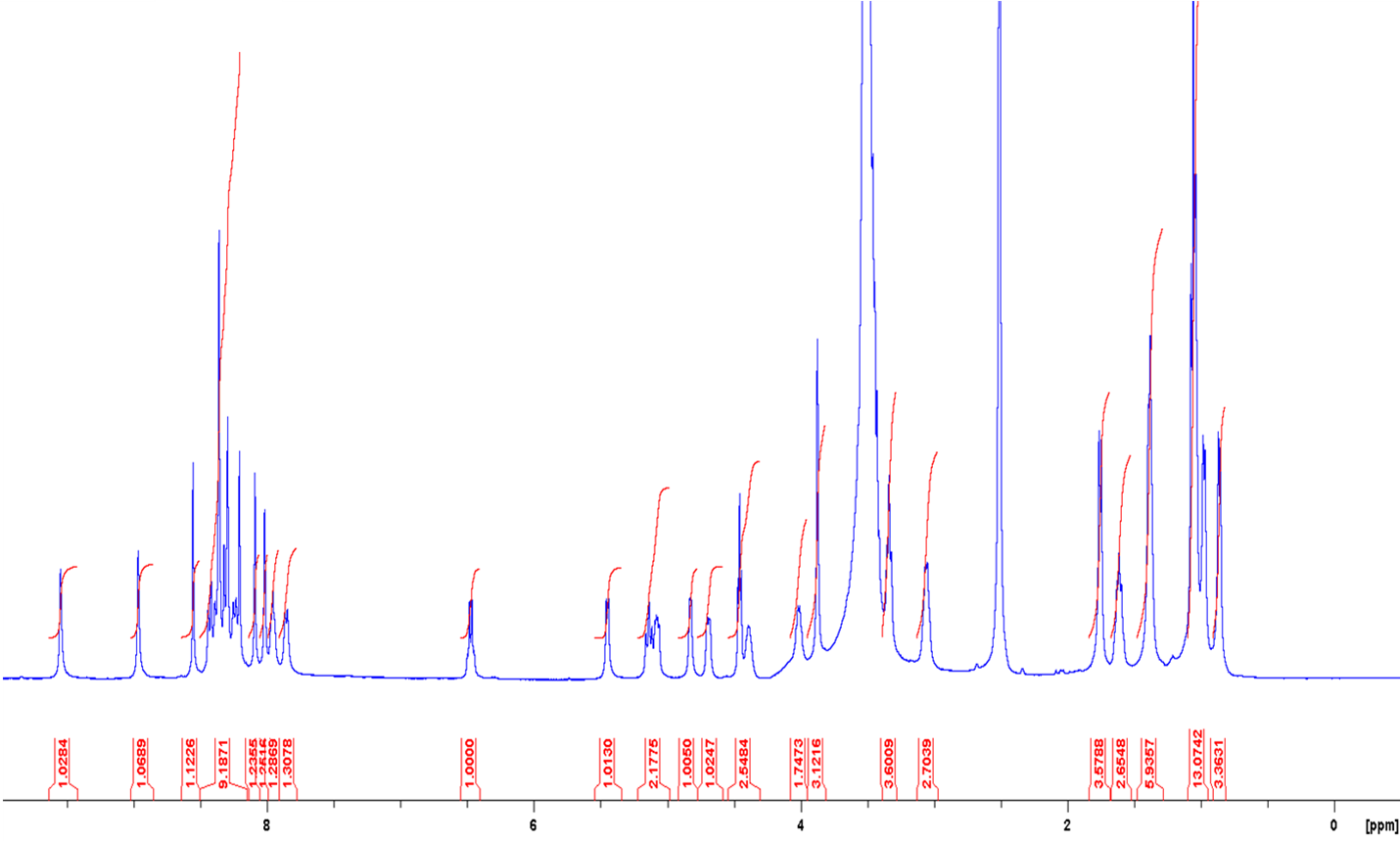


^13^C NMR


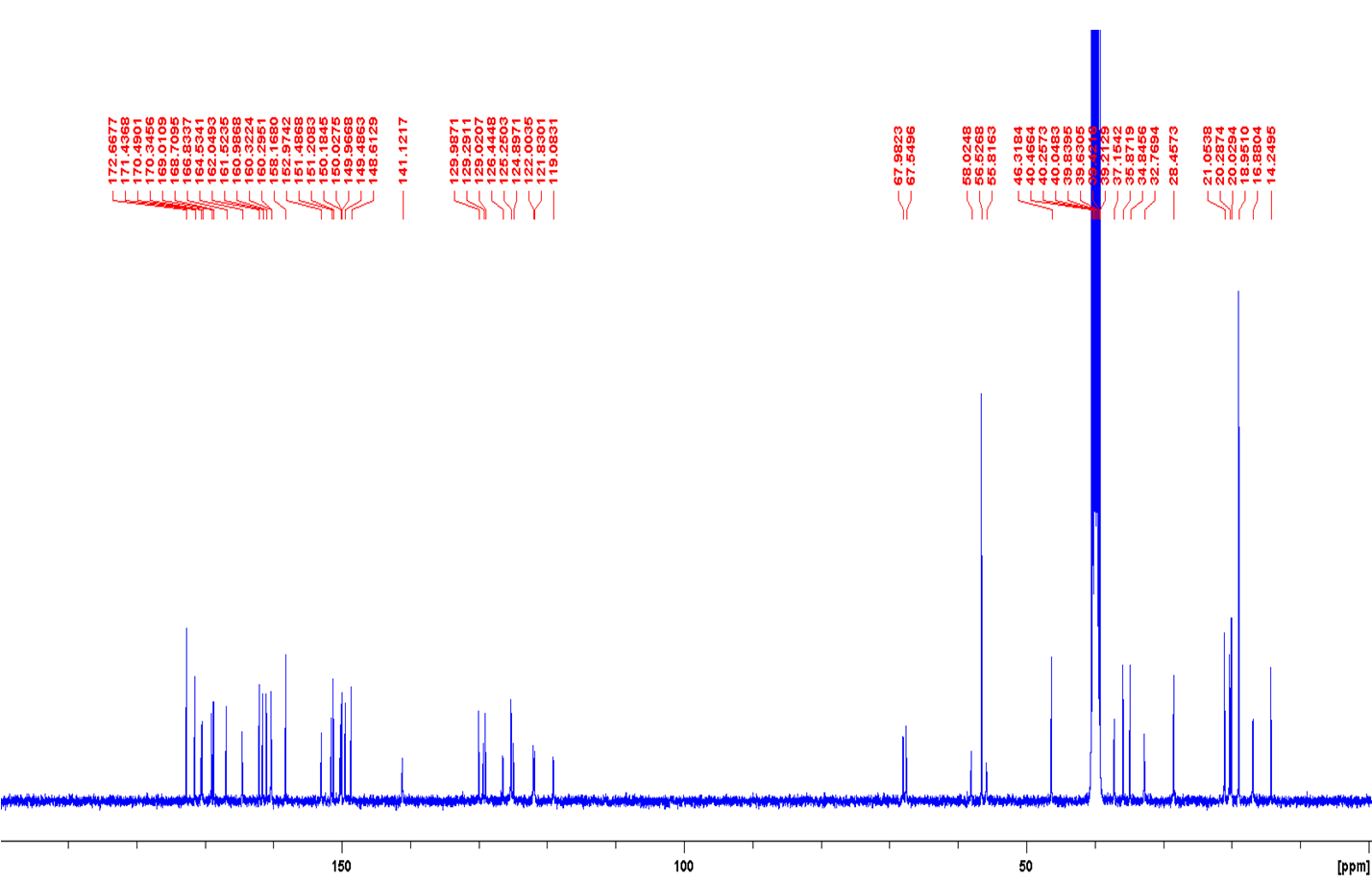


HPLC
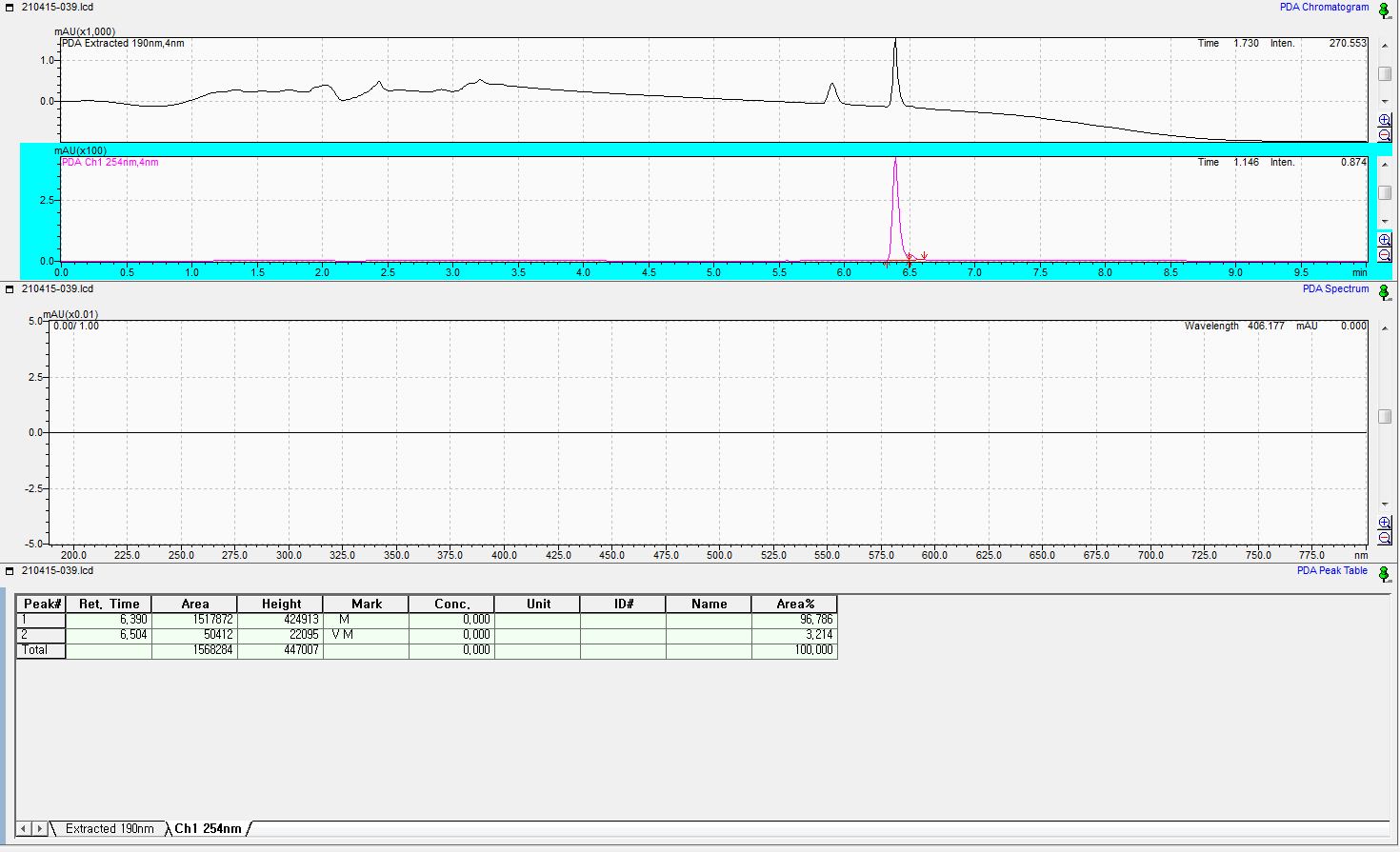


**Fig S2. ^1^H NMR, ^13^C NMR, HPLC and HRMS data of AJ-039**

**^1^H NMR (400 MHz, DMSO-*d6*):** δ 9.55 (s, 1H), 8.97 (s, 1H), 8.56 (s, 1H), 8.47 – 8.16 (m, 8H), 8.09 (s, 1H), 8.02 (s, 1H), 7.96 (s, 1H), 7.86 (d, *J* = 7.5 Hz, 1H), 6.47 (q, *J* = 6.6 Hz, 1H), 5.45 (d, *J* = 6.0 Hz, 1H), 5.14 (t, *J* = 9.0 Hz, 1H), 5.08 (dd, *J* = 8.2, 4.4 Hz, 1H), 4.83 (d, *J* = 3.5 Hz, 1H), 4.69 (d, *J* = 5.2 Hz, 1H), 4.46 (t, *J* = 5.0 Hz, 1H), 4.39 (s, 1H). 4.01 (bs, 1H), 3.87 (s, 2H), 3.34 (t, *J* = 6.6 Hz, 2H), 3.13 – 3.04 (m, 2H), 1.75 (d, *J* = 6.7 Hz, 3H), 1.65 – 1.51 (m, 2H), 1.39 (d, *J* = 5.6 Hz, 5H), 1.12 – 0.92 (m, 8H), 0.86 (d, *J* = 6.2 Hz, 3H).

**^13^C NMR (101 MHz, DMSO-*d6*):** δ 172.7, 171.4, 170.5, 170.3, 169.0, 168.7, 166.8, 164.5, 162.0, 161.5, 161.0, 160.3, 160.3, 158.2, 153.0, 151.5, 151.2, 150.2, 150.0, 150.0, 149.5, 148.6, 141.1, 130.0, 129.3, 129.0, 126.4, 125.3, 124.9, 122.0, 121.8, 119.0, 68.0, 67.5, 58.0, 56.5, 55.8, 46.3, 37.2, 35.9, 34.8, 32.8, 28.4, 21.1, 20.3, 20.0, 19.0, 16.9, 14.2.

**HRMS (FAB):** *m/z* calc for [C_51_H_51_N_15_O_10_S_6_+H]^+^ 1226.2340, found 1226.2341.

**HPLC:** flow rate 1.0 mL / min, t_R_ (5% -> 100 % acetonitrile / 95% -> 0% water (containing 0.1% TFA)) = 6.39 min, area 424913, purity 97%.

^1^H NMR


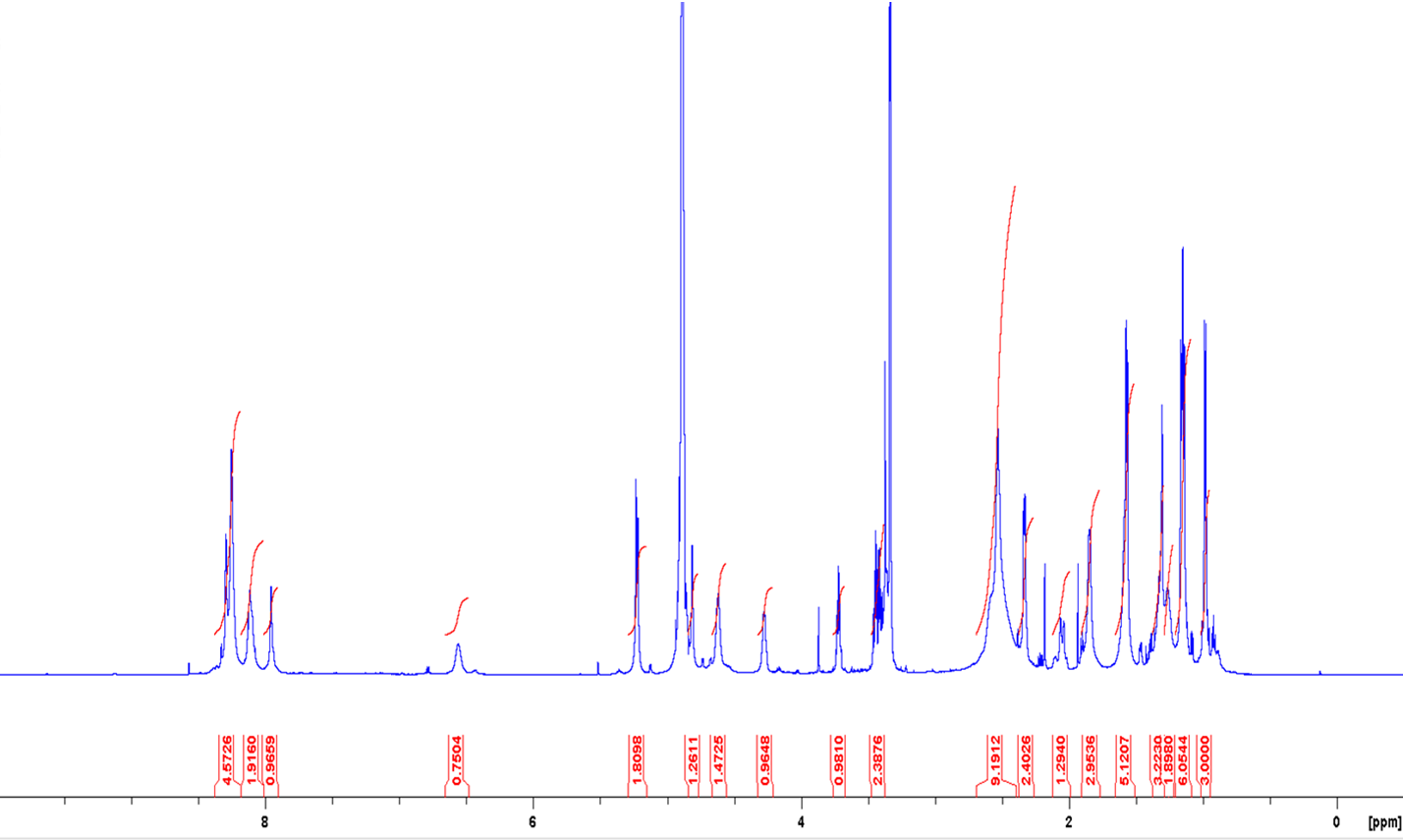


^13^C NMR


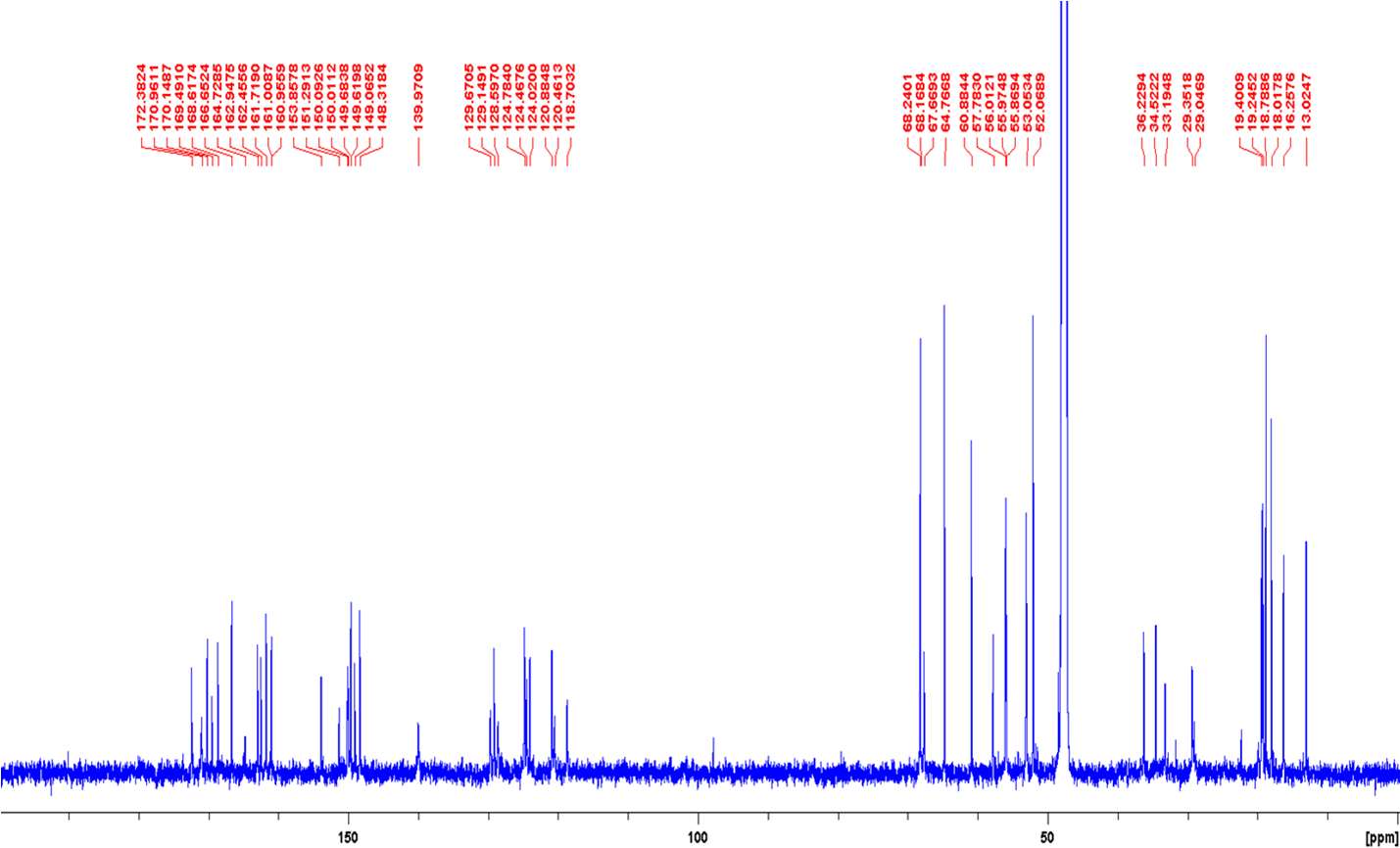


HPLC


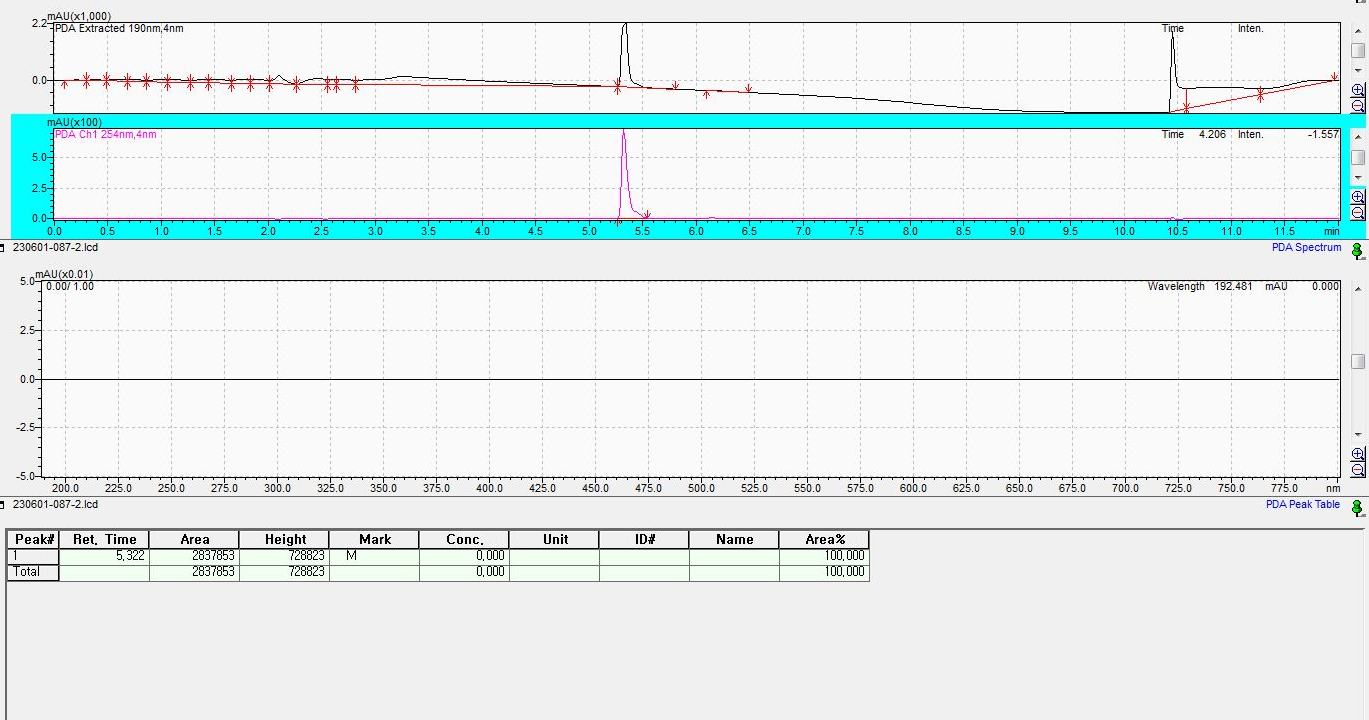


**Fig S3. ^1^H NMR, ^13^C NMR, HPLC and HRMS data of AJ-206**

**^1^H NMR (600 MHz, CD_3_OD):** δ 8.36 – 8.19 (m, 5H), 8.16 – 8.07 (m, 2H), 7.96 (s, 1H), 6.56 (bs, 1H), 5.24 – 5.18 (m, 2H), 4.81 (d, *J* = 2.1 Hz, 1H), 4.62 (bs, 1H), 4.27 (d, *J* = 3.7 Hz, 1H), 3.78 – 3.66 (m, 1H), 3.48 – 3.37 (m, 2H), 2.62 – 2.41 (broad band, 9H), 2.33 (d, *J* = 5.9 Hz, 2H), 2.13 – 1.97 (m, 1H), 1.91 – 1.75 (m, 3H), 1.63 – 1.52 (m, 5H), 1.39 – 1.29 (m, 3H), 1.27 – 1.21 (m, 2H), 1.20 – 1.07 (m, 6H), 0.98 (d, *J* = 6.6 Hz, 3H).

**^13^C NMR (151 MHz, CD_3_OD):** δ 172.4, 171.0, 170.1, 169.5, 168.6, 166.6, 164.7, 162.9, 162.4, 161.7, 161.0, 160.9, 153.8, 151.3, 150.1, 150.0, 149.7, 149.6, 149.1, 148.3, 140.0, 129.7, 129.1, 128.6, 124.8, 124.5, 124.0, 120.9, 120.5, 118.7, 68.2, 68.1, 67.7, 64.8, 60.9, 57.8, 56.0, 56.0, 55.9, 53.0, 52.1, 36.2, 34.5, 33.2, 29.3, 29.0, 19.4, 19.2, 18.8, 18.0, 16.2, 13.0.

**HRMS (ESI):** *m/z* calc for [C_54_H_61_N_15_O_10_S_6_+H]^+^ 1272.3122, found 1272.3137.

**HPLC:** flow rate 1.0 mL / min, t_R_ (5% -> 100 % acetonitrile / 95% -> 0% water (containing 0.1% TFA)) = 5.32 min, area 2837853, purity >99%.

**
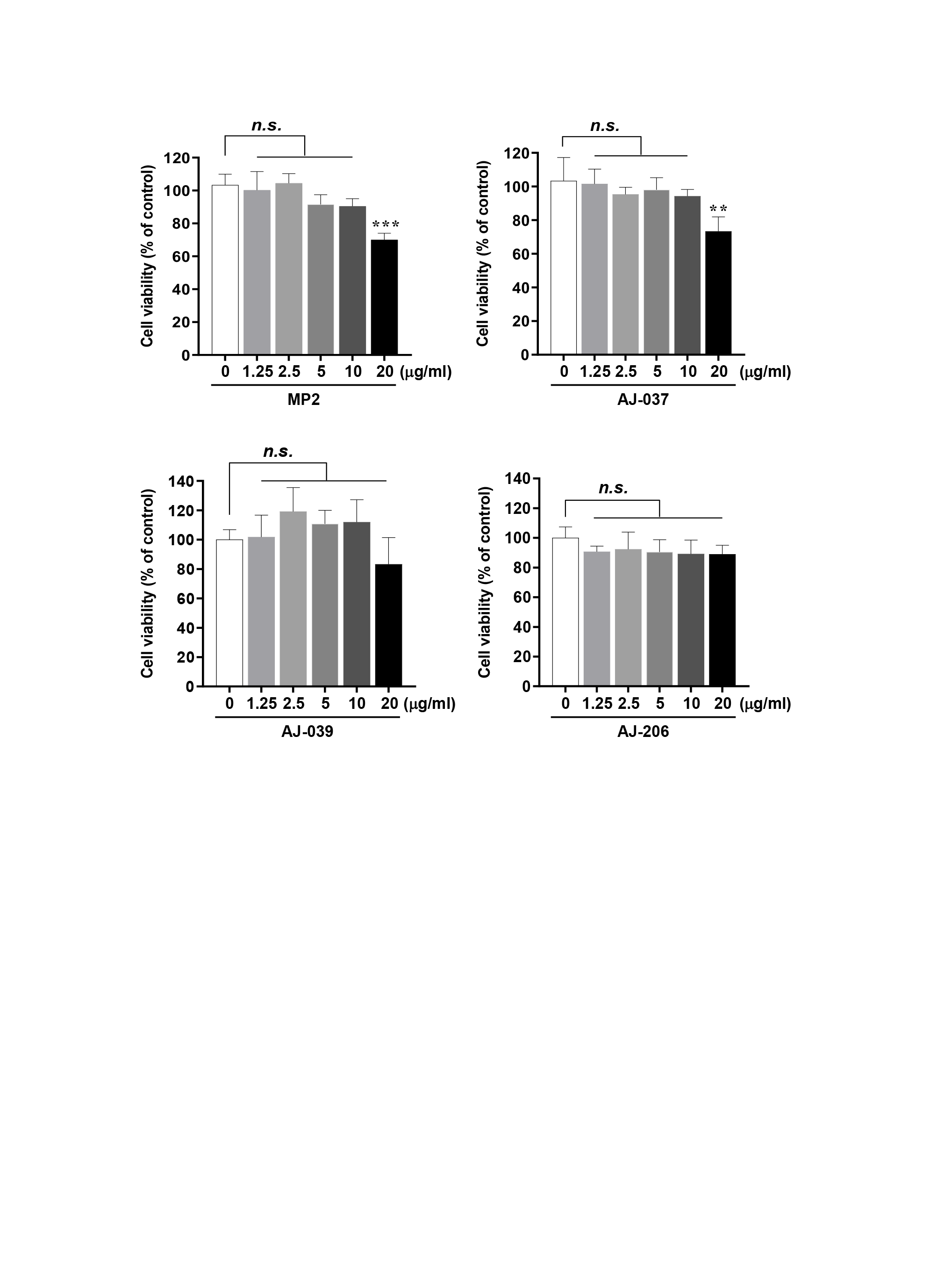
**

**Fig S4. Cell cytotoxicity of thiopeptide derivatives**

The cytotoxicity of thiopeptide derivatives was analyzed in BMDMs. Cell viability was presented as the percent of growth compared to control. The statistical significance was calculated by one-way ANOVA followed by Tukey’s multiple comparison test. The results are presented as the mean ± SD. **p* < 0.05; ***p* < 0.01; ****p* < 0.001; *n.s.*, not significant.


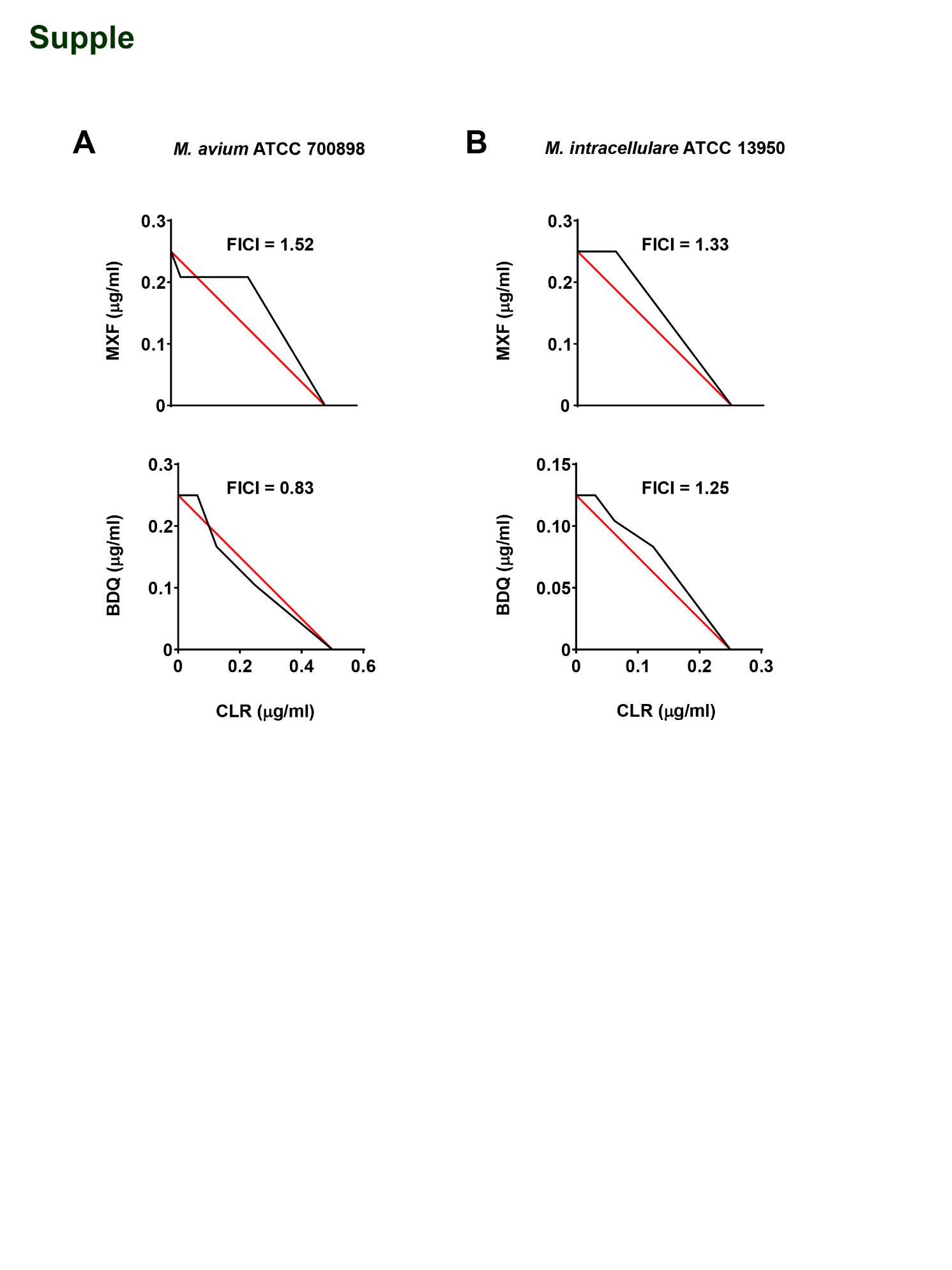


**Fig S5. Isobologram analysis of the checkerboard assay with CLR and other antibiotics against MAC strains**

Isobolograms of the combination of CLR with MXF and BDQ were created using a checkerboard assay against (A) *M. avium* ATCC 700898 or (B) *M. intracellulare* ATCC 13950. The red line indicates reference MICs of a single drug. The black line indicates the result from the checkerboard assay. The data are expressed as the average of three independent experiments.


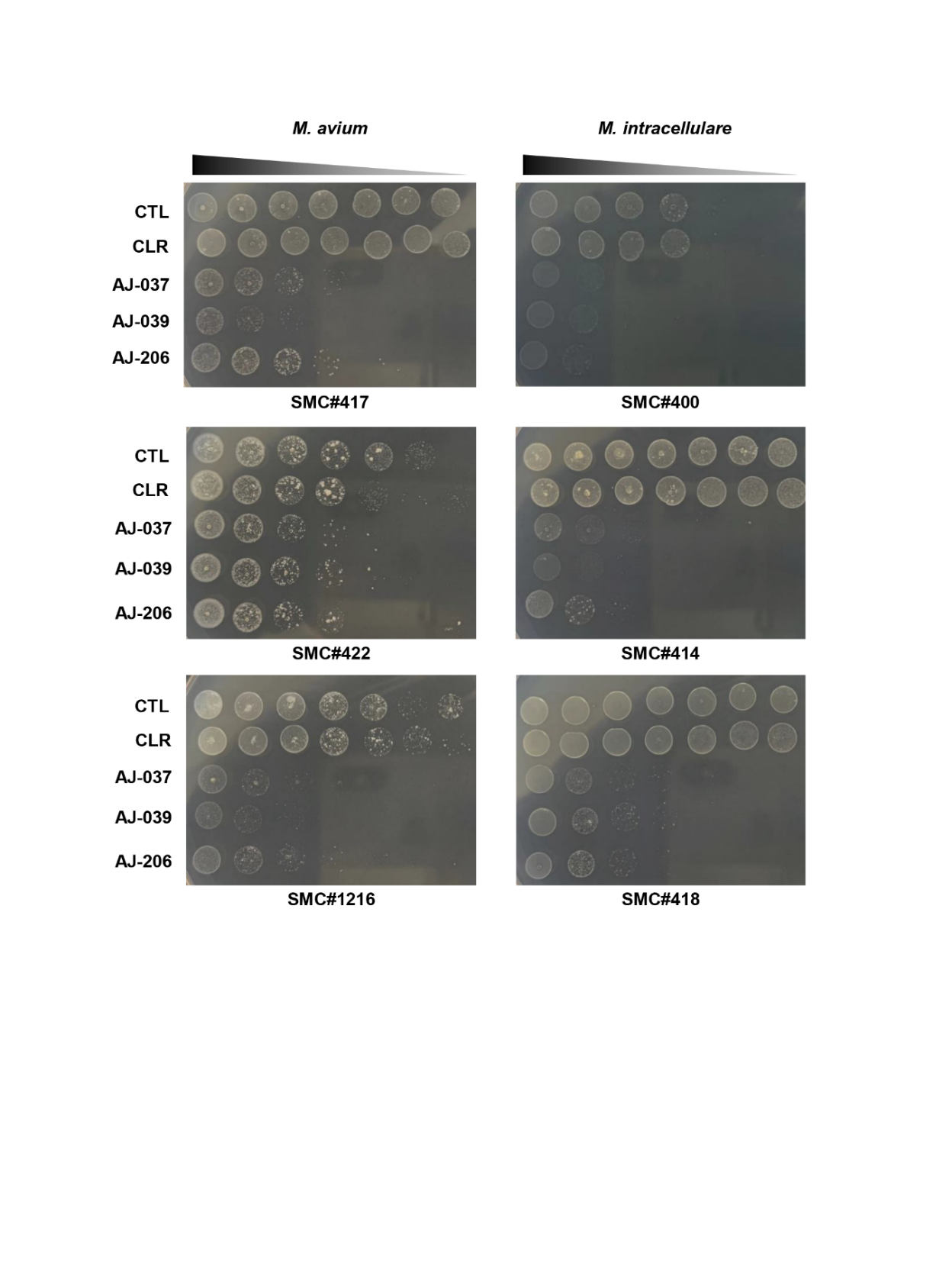


**Fig S6. Spotting growth assay of macrolide-resistant MAC strains with thiopeptide derivatives**

Spotting growth assays of serially diluted macrolide-resistant MAC strains were performed. Bacteria were serially diluted from 10^7^ CFU/mL to 10-fold, and growth was scored after 6 days. CLR and thiopeptide derivatives were administered at 64 μg/mL and 1× MIC.
